# Correlations between experiments and simulations for formic acid oxidation†

**DOI:** 10.1039/d2sc05160e

**Published:** 2022-10-26

**Authors:** Alexander Bagger, Kim D. Jensen, Maryam Rashedi, Rui Luo, Jia Du, Damin Zhang, Inês J. Pereira, María Escudero-Escribano, Matthias Arenz, Jan Rossmeisl

**Affiliations:** University of Copenhagen, Department of Chemistry Universitetsparken 5 2100 Kbh-Ø Denmark alexander@chem.ku.dk; College of Science, University of Tehran Enghelab Square Tehran Iran; School of Environmental and Biological Engineering, Nanjing University of Science & Technology Nanjing 210094 China; University of Bern, Department of Chemistry, Biochemistry and Pharmaceutical Sciences CH-3012 Bern Switzerland; Catalan Institute of Nanoscience and Nanotechnology (ICN2), CSIC, Barcelona Institute of Science and Technology UAB Campus, 08193 Bellaterra Barcelona Spain; ICREA Pg. Lluís Companys 23 08010 Barcelona Spain

## Abstract

Electrocatalytic conversion of formic acid oxidation to CO_2_ and the related CO_2_ reduction to formic acid represent a potential closed carbon-loop based on renewable energy. However, formic acid fuel cells are inhibited by the formation of site-blocking species during the formic acid oxidation reaction. Recent studies have elucidated how the binding of carbon and hydrogen on catalyst surfaces promote CO_2_ reduction towards CO and formic acid. This has also given fundamental insights into the reverse reaction, *i.e.* the oxidation of formic acid. In this work, simulations on multiple materials have been combined with formic acid oxidation experiments on electrocatalysts to shed light on the reaction and the accompanying catalytic limitations. We correlate data on different catalysts to show that (i) formate, which is the proposed formic acid oxidation intermediate, has similar binding energetics on Pt, Pd and Ag, while Ag does not work as a catalyst, and (ii) *H adsorbed on the surface results in *CO formation and poisoning through a chemical disproportionation step. Using these results, the fundamental limitations can be revealed and progress our understanding of the mechanism of the formic acid oxidation reaction.

## Abstract 2

Electrocatalytic conversion of formic acid oxidation to CO_2_ and the related CO_2_ reduction to formic acid represent a potential closed carbon-loop based on renewable energy.

## Graphical abstract

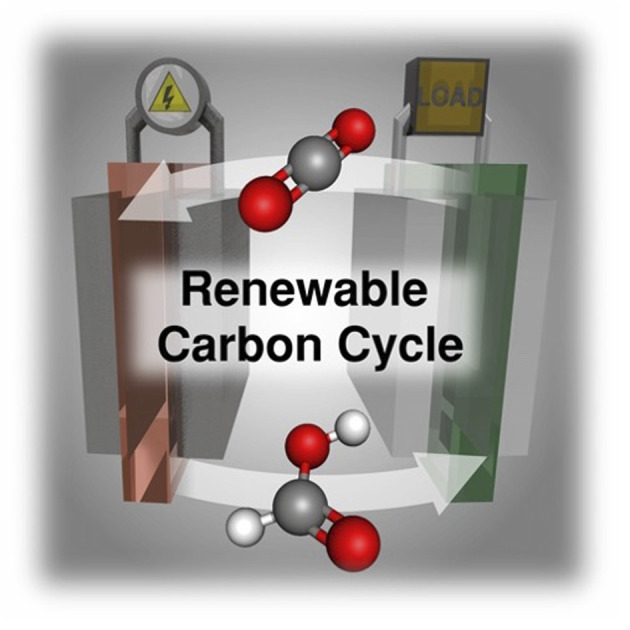

## Introduction

Tremendous efforts are currently going into out-phasing fossil fuels in favor of sustainable fuels.^1^ This is motivated by our need to close the carbon cycle^2^ and pave the way for new fuel production routes.^3^ Electrocatalytic technologies can in the future possibly allow direct electrification of chemical and fuel production. Examples include reduction of CO_2_ towards CO, HCOOH, C_2_H_4_, C_2_H_5_OH and H_2_O towards H_2_.^1^ Efficient fuel consumption through fuel cells (FCs) also holds great potential.^4^ Liquid fuels such as formic acid and methanol have attracted a lot of attention due to their viable energy density per mass- and volume, attractive handling/storage properties and potential uses in other non-fuel applications, *e.g.* as high value chemical building blocks for industry.^3^

Some liquid fuels, such as methanol, are notoriously limited in the oxidation toward CO_2_ since the process goes through a CO intermediate.^5^ CO oxidation then becomes the limiting factor determining the performance of direct methanol FCs (DMFCs). Formic acid as liquid fuel behaves differently; it has a CO_2_-like structure with two hydrogens attached. This molecular structure predicates that the oxidation process only requires the removal of two hydrogen atoms. Consequently, formic acid oxidation should ideally circumvent the problem of CO-poisoning.

To gauge formic acid's efficiency as a fuel we compare the single round trip efficiency of relevant closed-loop chemical compounds, *i.e.* hydrogen, formic acid, methanol and lithium batteries as seen in Table 1. Here we observe that the Li-battery storing and release of energy exhibits the highest efficiency followed by hydrogen. However, both Li-batteries and H_2_ suffer from low energy density. Storing energy as methanol and formic acid is very similar in terms of the cost in electrolyzer energy. The major difference between formic acid and methanol arises when using the chemical in a fuel cell, where methanol is limited by CO oxidation.^6^

**Table 1:** Estimated round-trip efficiencies 
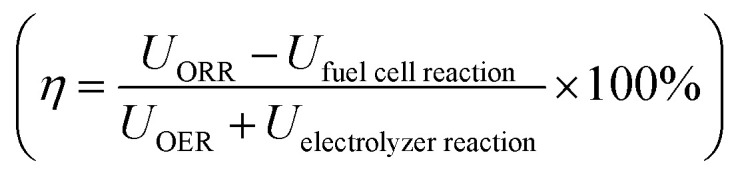
 calculated using the difference in energy potentials. Common for hydrogen, formic acid, and methanol we use *U*_OER_ = 1.6 V_RHE_ and *U*_ORR_ = 0.8 V_RHE_

	Energy stored (electrolyzer)	Energy released (fuel cell)	Round trip efficiency, *η*
Hydrogen	*U*_HER_ = −0.1 V_RHE_	*U*_HOR_ = 0.1 V_RHE_	≈41%
Formic acid	*U*_CO_2_→HCOOH_ = −0.8 V_RHE_	*U*_FAOR_ = 0.2 V_RHE_	≈25%
Methanol^a^	*U*_CO_2_→CO+H_2__ = −0.6 V_RHE_	*U*_CO→CO_2__ = 0.65 V_RHE_	≈7%
Li-battery^b^			≈90%

a Here only the cost of syngas production is considered, not the full formation of methanol.

b Typical charge/discharge efficiency.

Depending on the *U*_FAOR_, direct formic acid fuel cells (DFAFCs) can be considered an attractive alternative to methanol fuel cells. Methanol provides 6 protons per reacted molecule and formic acid only two and therefore methanol has a ∼3 times higher volumetric energy density. However, the potential of a formic acid fuel cell is high; even a few hundred millivolts reduction in overpotential can allow formic acid to output more energy than methanol per molecule.

Key to understanding the limitation of formic acid oxidation is the direct link to the reverse electrochemical reaction, *i.e.* the CO_2_ reduction reaction (CO_2_RR).^7,8^ CO_2_RR to formic acid and the formic acid oxidation reaction (FAOR) can be written in the form:1
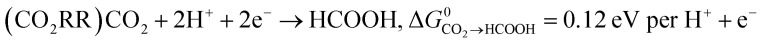
2
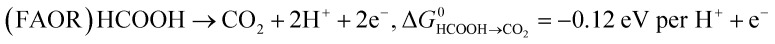
where Δ*G*^0^ is the thermodynamic potential per proton–electron pair of the reaction.

CO_2_RR selectivity is highly dependent on the catalyst material used and the crystal orientation.^9–12^ Hori *et al.* showed that hydrogen is produced on Pt, Ru, Fe and Ni, carbon monoxide is produced on Au, Ag, Zn, Ga and Pd (with limited amounts of H_2_), and finally, formic acid is produced on Pb, In, Hg, Sn, Cd and Tl with almost 100% faradaic efficiency.^11^ Importantly, hydrocarbons are uniquely produced on Cu.^11^ Using simulations, we were able to classify the CO_2_RR product distributions towards hydrogen, hydrocarbons, CO and formic acid due to the catalyst's affinity towards adsorbed *H and *CO.^10^ Interestingly, we noted that the CO_2_RR appears selective towards formic acid when weakening *H binding (*i.e.* when there is no *H on the catalyst surface). Moreover, from this study^10^ we noted that the *OOCH *vs.* the *COOH intermediate cannot be used to distinguish the CO or formic acid product formation. Where previous works used *OOCH as a descriptor for formic acid oxidation and *COOH as a descriptor for CO production, and even recent discussion for CO_2_ reduction highlights the possible formic acid formation through *COOH.^13^ These findings and discussion are readily usable when considering the reverse reaction FAOR, which involves similar reaction intermediates to CO_2_RR and *vice versa*.

FAOR exhibits the highest intrinsic activity on Pt and Pd.^14–19^ However, the reaction is affected by high overpotential and formation of various poisoning and site-blocking intermediates.^20^ The following observations are reported in the literature, as also shown in ESI Fig. S1:† (i) FAOR onsets at low potentials does not necessarily correspond to high FAOR currents. (ii) Hysteresis between anodic and cathodic scans is a common occurrence, showing a higher current in cathodic scans in than anodic ones, which shows that the reaction rate depends on the prehistory. Typically, this “memory effect” is related to unwanted reactions which form species that block the surface, *i.e.* poisoning species. (iii) Pt(111) is more active than Pd(100) in the low overpotential region; however, interestingly, this relationship shifts at higher potentials. (iv) As an observation it is known that there is a difference for Pt and Pd with respect to the CO poisoning during FAOR.^8^ The ideal FAOR catalyst on the other hand should show reversible cyclic voltammetry (CV), high activity, low onset potential and stable currents, as illustrated in Fig. S2.†

**Fig. 1:**
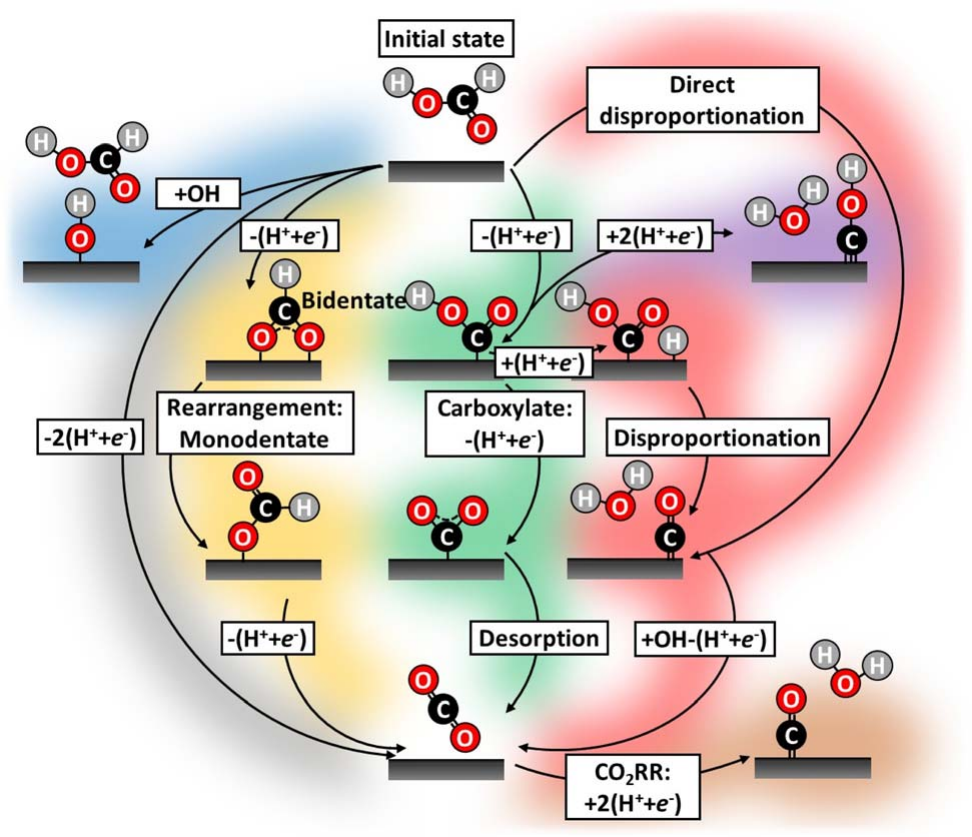
Literature study highlighting all the conceived FAOR reaction pathways during potential cycling.^38^ Historically, FAOR has been split into the direct (gray)^39,40^ and indirect (green and yellow)^16,39,41,42^ pathways. Further, partial FAOR and catalyst oxidation forming unwarranted surface blocking have been suggested, *e.g.* CO_*x*_H_*y*_ species (purple),^20,25,43^ CO (red)^44^ and hydroxide/oxides (blue).^38^ Even CO_2_RR induced CO formation by applying too cathodic potential (brown)^40^ have been suggested. Recently, formate (yellow)^28,39,45,46^ in various arrangements has gained attention as potential catalyst site-blocking agents.

To understand the mechanism of this important reaction numerous attempts have been made to map possible FAOR pathways,^20–24^ which we illustrate by the literature study overview in Fig. 1. A dual-pathway mechanism for formic acid oxidation is established by the community:^25,26^ The direct pathways, which leads to the desired final product of CO_2_ through the formate adsorption,^27^ and another path where adsorbed CO, the poisoning species, is formed. The CO formation can be thought of as being formed through a so-called chemical disproponation reaction, where an activated formic acid intermediate reacts with a hydrogen to form water and CO (*e.g.* *COOH + *H → *CO + H_2_O). However, the nature of the reactive intermediate in the direct pathway is still under strong debate and it is not given that it is the most stable intermediate (*e.g.*, formate) should also be the reaction intermediate. The community has focused on elucidating the reaction mechanism and attempted to circumvent poisoning issues: through pathway engineering,^21,22^ changing electrolyte composition,^23^ or inclusion of sites with the ability to remove poisoning or site-blocking species.^24,28–30^ CO-poisoning from partial HCOOH oxidation is often considered the principal culprit^28^ and various works suggest CO formation can be avoided utilizing a single/dual-site catalyst.^31–34^ However, catalysts such as Au–Pt,^21^ Pt–Hg/C^35^ and Pd–Hg/C^36^ exhibit limited catalytic improvement over their pure metal counterparts, for some overviews see Fig. S4 in ESI.† In this work, we will take a new view on all possible pathways for working catalysts. We carry out electrochemical characterization on Pd/C and Pt/C catalyst as well as Pt–Hg/C,^35^ Pd–Hg/C^36^ and Pt–Bi/C,^37^ following the protocols reported in the literature for the preparations of these catalysts. Furthermore, we try to validate the possible reaction path for not/low performing catalysts (*e.g.*, Ag) for formic acid oxidation.

In this work, we address the following fundamental questions in FAOR:

(i) Is the FAOR activity correlated with the *COOH or the *OOCH intermediate?

(ii) How is CO formed during the FAOR?

To probe the scientific questions, we use a combination of experimental tools based on cyclic voltammetry (CV) and chronoamperometric (CA) and for simulations we use density functional theory (DFT) calculations on the binding of carboxyl, *COOH, formate bidentate, *OOCH, and hydrogen, *H.

## Results and discussion

**Fig. 2:**
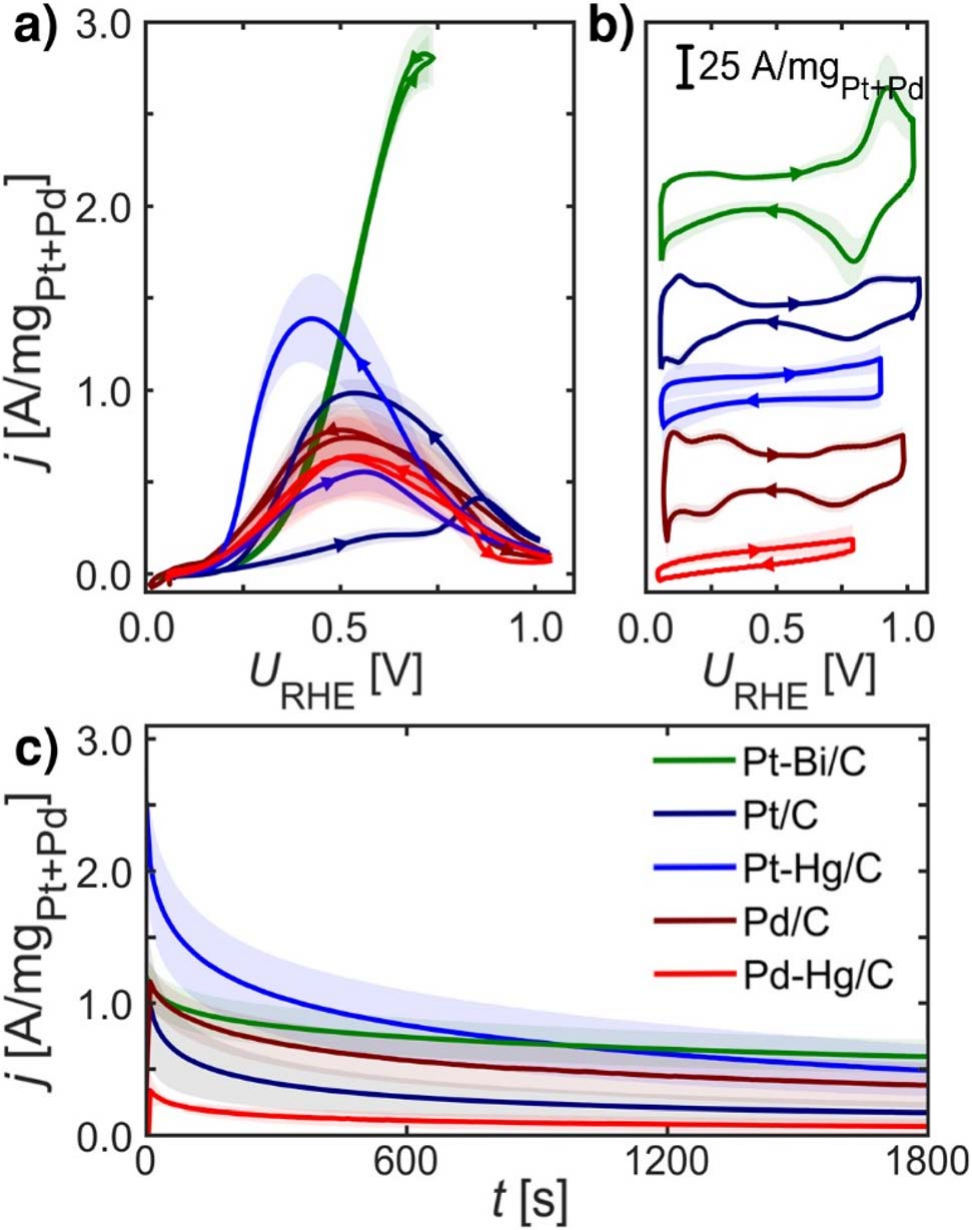
Electrochemical data recorded at room temperature of equal loading nanoparticle catalysts: Pt/C (navy blue), Pd/C (crimson), Pt–Hg/C (blue), Pd–Hg/C (red) and Pt–Bi/C (green) systems on glassy carbon in Ar-saturated 0.1 M HClO_4_ with 0.1 M HCOOH at 1600 rpm, unless otherwise stated. (a) FAOR CVs at 10 mV s^−1^. (b) Base CVs in Ar-saturated 0.1 M HClO_4_ at 400 rpm and 50 mV s^−1^. (c) CA at 0.55 V_RHE_ for 30 min. All measurements were repeated at least three times (shade represents standard deviation), *IR*-compensated and post-corrected, for experimental details see ESI and Fig. S5–S8.† Note, Pt–Bi/C was unstable at higher potentials (see ESI†) hence a reduced potential range was utilized.

Fig. 2 summarizes experimental electrochemical data of Pt/C, Pd/C, Pt–Hg/C, Pd–Hg/C and Pt–Bi/C. Here we investigate only known and active FAOR catalyst, and both extended surfaces as Pt/C and Pd/C, but also single site catalyst Pt–Hg/C and Pd–Hg/C and the noteworthy very active Pt–Bi/C system. These investigated catalysts are synthesized from the same starting materials, *i.e.* premade Pd or Pt catalyst which we then modified by (electro)deposition of Hg or Bi at room temperature. Consequently, it is reasonable to expect that no significant impact of the catalysts' surface area arises due to the modification, allowing for catalyst comparison. However, verifying this expectation using standard electrochemical surface area (ECSA) evaluation (*e.g.* utilizing *CO-stripping or hydrogen under potential deposited, H-UPD, charges) is not possible as these species do not adsorb specifically on Hg and Bi modified catalysts.

Fig. 2a shows the formic acid oxidation CV during rotation and 10 mV s^−1^ scan-rate on the five catalysts. Fig. 2b depicts the base CVs exhibiting suppressed hydrogen underpotential deposition (H_UPD_) on the Pt–Bi/C, Pt–Hg/C and Pd–Hg/C compared to the Pd/C and Pt/C counterparts. Fig. 2c displays the formic acid oxidation CA at 0.55 V_RHE_ for 30 min, illustrating the loss in activity at this potential due to the formation of poisoning or blocking species. For Pt/C and Pt–Hg/C, an apparent hysteresis is seen in the oxidation between the forward and backward scans of Fig. 2a indicating an irreversible change in the catalyst going to low potentials. Interestingly, taking a combined view on Fig. 2a and c shows that forming single-sites of Pt through Hg alloying^35,36^ tend to improve the FAOR onset. In contrast, Pd-based catalysts generally do not exhibit any hysteresis. The Pt–Bi/C system exhibit the highest FAOR current with least hysteresis, but also with the highest overpotential. Additional relevant electrochemical studies can be found in the ESI,† represented through Fig. S10–S13.† To explain these observations in Fig. 2 we turned to DFT.

**Fig. 3:**
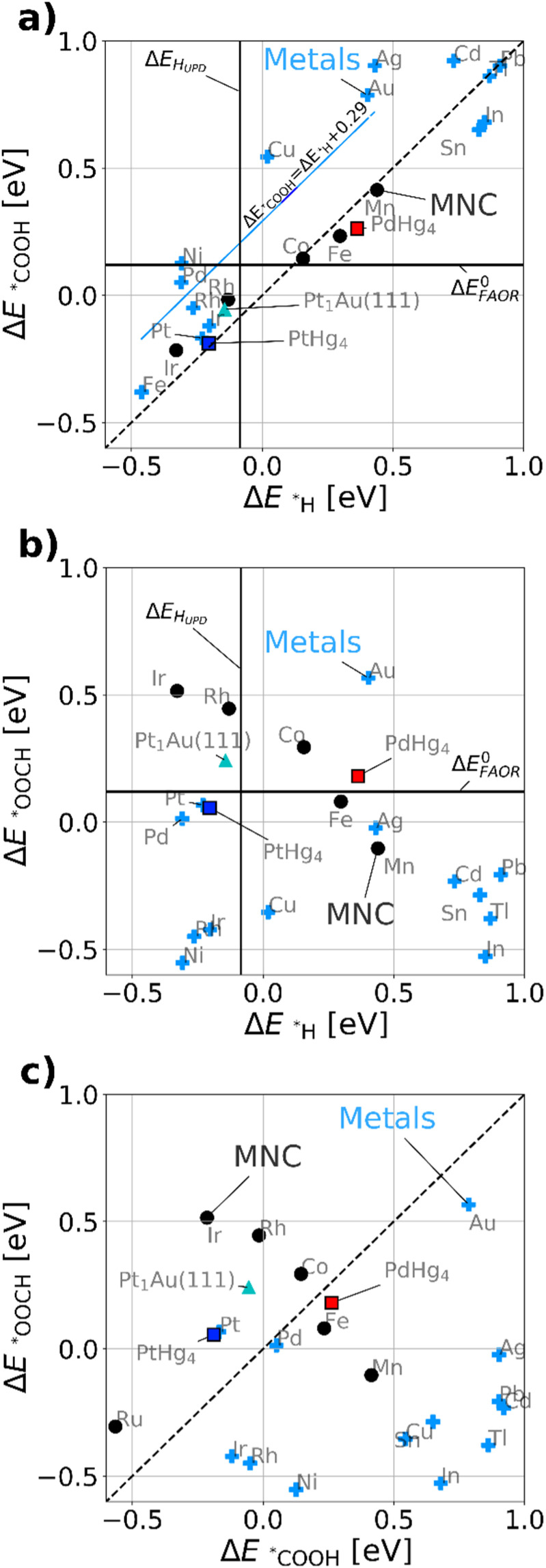
DFT calculated binding energies for metal fcc(111) (light-blue crosses) and single-site catalyst. MNC-based (black points), Pt atom in Au(111) denoted Pt_1_Au(111) (cyan triangle), PdHg_4_ (black/red square) and PtHg_4_ (black/blue square). (a) *COOH *vs.* *H. (b) *OOCH *vs.* *H. (c) *OOCH *vs.* *COOH, here the dashed line shows the diagonal indication the affinity towards formate bound through carbon or oxygen. Here its assumed Δ*E*^0^_FAOR_ ≈ Δ*G*^0^_FAOR_. Further, we used CO_2_ and H_2_ for references when calculating *COOH, Δ*E*^0^_FAOR_ is 0.12 eV per electron.

Fig. 3a maps out the DFT calculated *COOH *vs.* *H binding energies of the most relevant model metal (111) facets and single-site catalysts, such as MNCs, PtHg_4_,^35^ PdHg_4_ (ref. 36) and single Pt atoms in Au, Pt_1_Au(111)^21^ (for computational details see ESI†). *Via* simulations we study both metals and the single-site-catalysts as they both have a linear scaling: ΔE_*COOH_ = ΔE_*H_ + *b*. However, *b* is about 0.29 eV as previously^47^ observed for metals and 0.0 eV for single-site catalysts, respectively. This allows for a fundamental destabilization of *H *vs.* *COOH at the single-site catalyst motifs as compared to metals, which is beneficial for improved FAOR by limiting the disproportionation reaction. Besides the scaling, a vertical- and a horizontal line indicating H_UPD_ (Δ*E*_H_UPD__ = 0 for 1/2H_2_ ↔ *H) and formic acid's thermodynamic equilibrium potential have been included in Fig. 3a and b. Here its assumed that Δ*E*^0^_FAOR_ ≈ Δ*G*^0^_FAOR_, as thermodynamic corrections and water stabilization are expected to cancel out for the intermediates. The challenge to understand the implications of Fig. 3a is that one needs to compare *H as a reduction from H_2_O and *COOH as oxidation from HCOOH. This means that *H stabilizes with negative potential, while *COOH stabilizes with positive potential during FAOR. Essentially, from Fig. 3a it can be inferred that materials to the left of approximately Δ*E*_H_UPD__ are limited by disproportionation at low potentials. While materials to the right are limited by weak binding *COOH. Most importantly, the fundamental scaling between *COOH and *H matter as adsorption of *H on the surface leads to the possibility of disproportionation towards CO. In conclusion, the single-atom catalyst scaling crosses the FAOR thermodynamic potential at values significantly above Δ*E*_H_UPD__, which suggests that single-atom catalyst may work for FAOR near the FAOR equilibrium potential without carrying out the disproportionation reaction.

**Fig. 4:**
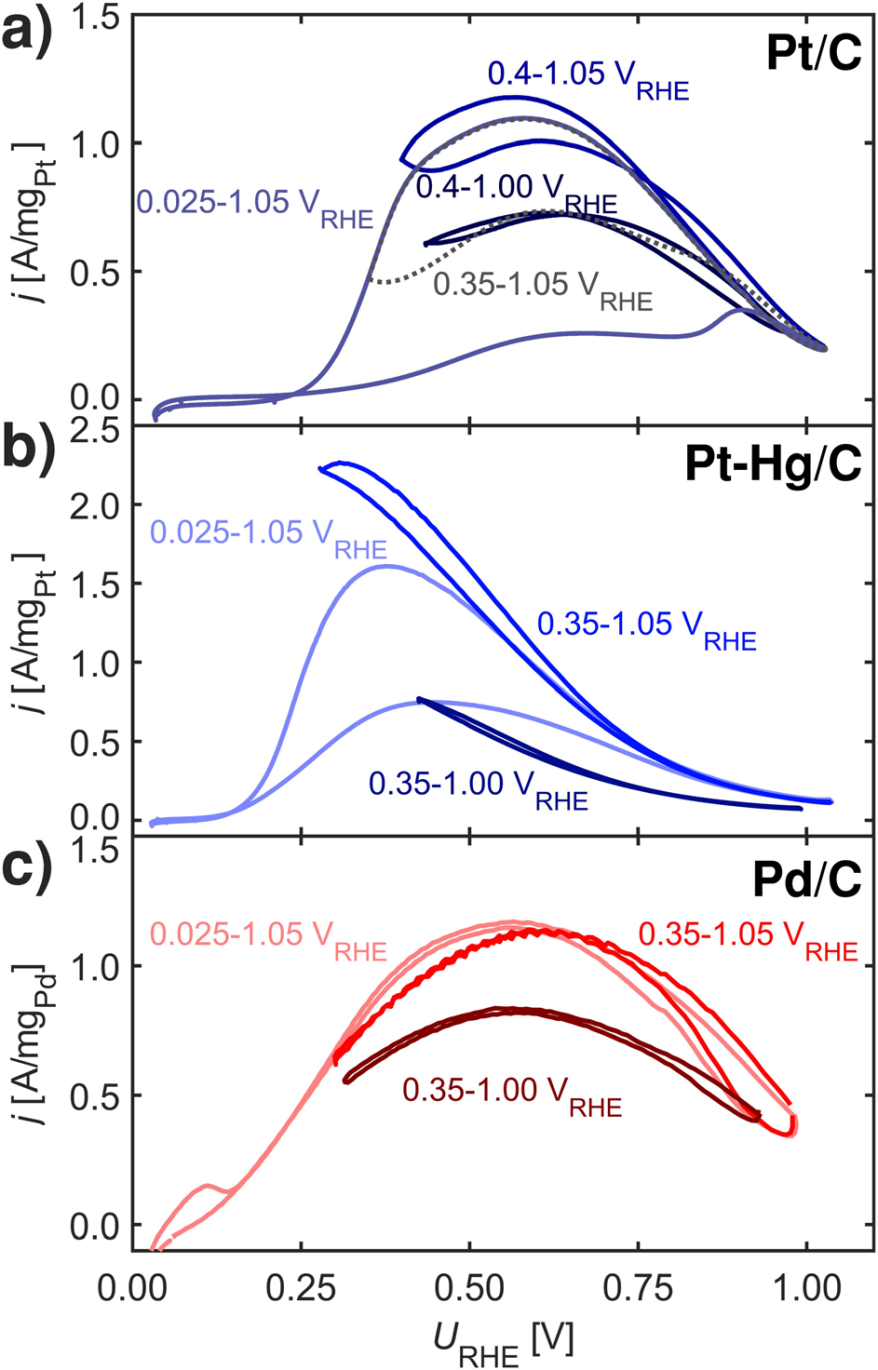
FAOR CVs at 10 mV s^−1^, 1600 rpm and room temperature in Ar-saturated 0.1 M HClO_4_ with 0.1 M HCOOH at different potential limits. Ohmic drops were post-corrected. (a) Pt/C. (b) Pt–Hg/C. (c) Pd/C. Note, increasing lower potential limit minimizes the hysteresis in the CVs, lowering the upper potential limit generally decreases the activity. Note, that after cycling in the different potential limits returning to the full range of 0.025 to 1.05 V_RHE_ re-initializes the FAOR response *i.e.* no irreversible changes arise due to dissolution or sintering.

Fig. 3b displays the DFT calculated binding energies of *OOCH *vs.* *H. Conversely to Fig. 3a, there is no apparent scaling between formate bidentate and adsorbed hydrogen. However the oxygen bond for *OOCH scales with *OH and (bi)-carbonate (HCO_3_/CO_3_), which have surface binding through oxygen. Carbonate species could be formed over time due to the equilibrium with CO_2_ and hence poisoning the surface, revealing a decaying activity as observed in Fig. 2c for all working FAOR catalysts. Investigating *OOCH as a function of *H is important as *OOCH has been suggested as an important reaction intermediate. Fig. 3b shows that the binding energy of the *OOCH intermediate on Ag is slightly too strong, but very close to the thermodynamic potential and similar to Pt and Pd. Essentially, if *OOCH was the important reaction intermediate for FAOR, then Ag should be working as FAOR catalyst at very low potential, and furthermore Ag does not suffer from disproportionation (as Ag *H binding far exceeds Δ*E*_H_UPD__). However, Ag is experimentally shown to be inert for the FAOR. The role of formate in the reaction is clearly a puzzle. For FAOR surface-enhanced IR absorption on Pt has experimentally confirmed formate on the surface^27^ above 0.7 V_RHE_ and further for CO_2_RR *in situ* surface-enhanced Raman spectroscopy on Ag has experimentally confirmed formate on the surface.^48^ As Pt converts formic acid to CO_2_ and Ag converts CO_2_ to CO, there seems no relation between the experimental observation of formate on the surface as reaction intermediate. We note that *OOCH scales with *OH, the *OOCH binding can be considered a probe of the oxidation affinity of the catalyst, *i.e.* having a strong *OOCH binding results in a lower oxidation potential.

Fig. 3c shows the *OOCH *vs.* *COOH binding for the catalyst, with a dashed line indicating the affinity towards carbon or oxygen. Depending on the catalyst we can see whether formate bidentate or carboxyl is favored. The working catalyst is Pt, which is well above the diagonal and Pd which is at the diagonal, while poorly working FAOR catalysts *e.g.* Ir^49^ or Au^50^ are slightly below the diagonal. Catalysts having stronger *OOCH binding *vs.* *COOH (below the diagonal), are basically oxidized before they can carry out FAOR.

The type of analysis illustrated in Fig. 3 is a powerful tool able to identify which catalyst suffers from disproportionation, poisoning or oxidation. By virtue of the *COOH *vs.* *H scaling-relations,^10^ it gives fundamental insights into why literature historically has shown no significant FAOR activity at the thermodynamic potential for a metal catalysts. Implicitly Fig. 3a shows disproportionation occurring at low potential arising from the H_UPD_ and the consequent creation of *CO poisoning species. The creation of *CO leads to hysteresis between the anodic and cathodic sweeps in FAOR CVs. From this insight, one would expect that disproportionation is mitigated in the CVs by simply staying above H_UPD_ potentials.

**Fig. 5:**
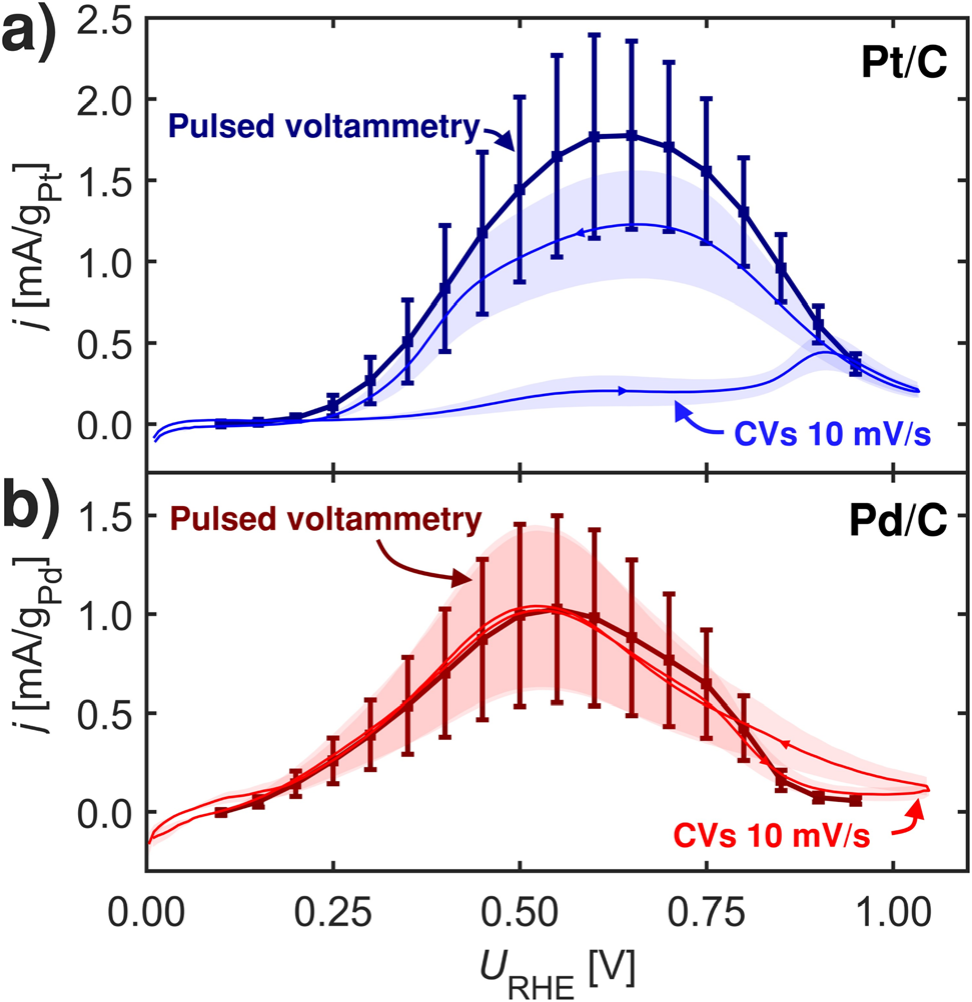
2 s pulsed voltammetry and CVs at 10 mV s^−1^ at room-temperature at 1600 rpm in Ar-saturated 0.1 M HClO_4_ with 0.1 M HCOOH. (a) Pt/C. (b) Pd/C. Measurements repeated three times.

Fig. 4 shows FAOR CVs of Pt/C, Pt–Hg/C and Pd/C cycled with varying potential limits. The potential ranges from 0.00–1.05 V_RHE_ reveals that Pt/C and Pt–Hg/C are poisoned in anodic sweeps. While, changing to potentials, ranging from 0.40–1.05 V_RHE_ for the Pt/C and 0.35–1.05 V_RHE_ for the Pt–Hg/C, significantly increases the anodic activity. Hence, this allows us to indicate that H_UPD_ mediated disproportionation account for poisoning through CO on Pt catalysts. For Pd/C in Fig. 5c, decreasing the lower potential limit has no influence on the almost non-existing FAOR hysteresis. In this context, it is important to note that Pd is well-known to form Pd-hydride phases^51^ below 0.2 V_RHE_, which then competes with *H adsorbed, *i.e.* at potentials relevant for both FAOR and CO_2_RR. In relation to CO_2_RR, we also note that Pd-hydride, leads to a high faradaic efficiency towards formate,^52^ whereas at higher overpotentials CO and H_2_ will dominate.^11^

Concerning Fig. 4, one could erroneously assume that lowering the upper potential limit would not affect the CVs, while staying above the CO oxidation potential. This is however not the case, cycling 50 times from 0.35–1.00 V_RHE_ reveals some form of deactivation of both Pt/C, Pt–Hg/C and Pd/C. We do not know what the origin of this deactivation is. Various studies reported in the literature suggests different reasons including deactivation to irreversible metal oxidation^53,54^ due to insufficient surface reduction or accumulation of either *OOCH,^55^ *COH,^38^ *OCOH^56^ or *CO species. Interesting is that *in situ* Fourier-transform infrared spectroscopy (FTIR) work^57,58^ has shown that Pt, contrary to Pd, continuously form CO above H_UPD_ potential during FAOR.

Finding that *H limits FAOR activity at low potential *via* disproportionation, allows one to hypothesize about the Pt–Bi and Pt–Hg systems, which perform better than Pt on two different perspectives. Pt–Bi shows no hysteresis and is active at higher overpotential as compared to Pt. The absence of a hysteresis in the CV indicates that Pt–Bi somehow circumvent the disproportionation reaction, potentially by blocking the surface for *H at low potential as onset for Pt–Bi is higher than on Pt. Pt–Hg on the other hand is more active in the negative sweep, particular at lower potential, but does show clear hysteresis. Further we hypothesize that the Pt single-sites in Pt–Hg destabilizes *H shifting the onset to lower potentials and increasing the activity at low potential, simply due to a limited disproportionation reaction.

To gauge how the FAOR is affected in the potentials regions above H_UPD_ we conducted pulsed voltammetry inspired by Clavilier *et al.*^59^ In this type of pulsed voltammetry experiments, each potential investigated is separated by a surface re-initialization (at 1.05 V_RHE_) cleaning the surface for all poisons through surface oxidation. The impact from dissolution at this oxidizing potential should be minimal.^60,61^

Fig. 5 shows pulsed voltammograms and corresponding CVs for Pt/C and Pd/C samples. Interestingly Pt/C becomes more active when pulsing, which is in contrast to Pd/C that does not show any changes from the pulsing. This experiment challenges *OOCH species as site-blocking species. Since Pd has stronger relative *OOCH to *COOH binding than Pt, and it should hence be on Pd where activity was affected by blocking of *OOCH species. One view for this observation could be given in the recent work by Koper and coworkers,^8^ who noted that formate adsorption is important for formic acid oxidation, not as an active intermediate, but more as a self-protector against CO poisoning.

**Fig. 6:**
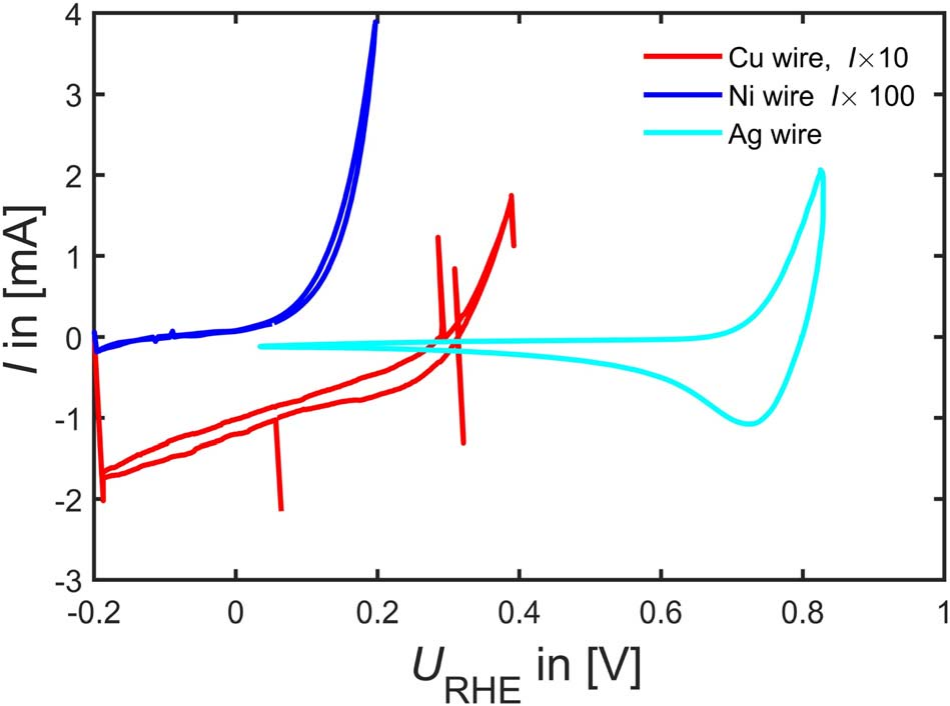
FAOR data on a Cu, Ni and Ag wire at 50 mV s^−1^ at room-temperature Ar-saturated 0.1 M HClO_4_ with 0.1 M HCOOH taken at 50 mV s^−1^.

Beyond Pd and Pt catalyst, in Fig. 6, we tested Cu, Ni and Ag wires as FAOR catalyst. The experiments revealed that Ni indeed seems very active to oxidize either HCOOH or Ni. However, going to potentials above 0.2 V_RHE_ tended to bring out a yellowish tinge in the electrolyte, and by looking into Ni's Pourbaix diagrams^62^ it appears to readily dissolve as Ni^2+^ at 0.15 V_RHE_ at pH 1, consequently making it a poor FAOR catalyst (unless there is some very narrow window that Ni is stable enough to oxidize HCOOH without dissolving). Similarly, Cu shows no FAOR activity only the well-known Cu oxidation current at potentials larger than 0.2 V_RHE_ is observable. Ag is perhaps active towards FAOR but again this occur close to Ag dissolution potentials suggesting it to be a rather poor FAOR catalyst.

Most interesting when combining the observation of similar intermediate binding energetics in Fig. 3, we identified that Ag has similar binding energetics of formate as Pd and Pt, whereas Cu and Ni do not follow the energetic of Pd and Pt. Hence Cu and Ni can be used as test catalysts far from known working catalysts. However, as tested here in Fig. 6 there appears no significant FAOR on Ag, Cu and Ni.

## Conclusion

In conclusion we have correlated the FAOR activity with simulated DFT binding energies of *COOH, *OOCH and *H across multiple metal catalysts. We have observed that for an ideal catalyst, the FAOR equilibrium potential should be above its corresponding H_UPD_ potential in order to avoid the disproportionation. We found that *COOH and *H binding scale on both metal and single-site catalysts. This creates the fundamental limiting potential due to H_UPD_ mediated disproportionation on the surface. The carbon–hydrogen scaling is indeed a fundamental limitation, analog to the *OH and *OOH scaling for oxygen evolution and reduction. Experimentally, we show that a good performing FAOR catalyst should have the attributes of: (i) an onset close to the fundamental derived onset, (ii) no hysteresis between anodic- and cathodic CV scans and (iii) high and stable FAOR CA currents above the derived onsets fundamental limits. Interestingly, this works concludes on the direct relation between FAOR and CO_2_RR; *H in combination with *COOH forming CO in both FAOR and CO_2_RR.

## Abbreviations

CA: Chronoamperometry
CV: Cyclic voltammogram/voltammetry
CO_2_RR: CO_2_ reduction reaction
DFAFC: Direct formic acid fuel cell
DMFC: Direct methanol fuel cell
DFT: Density functional theory
fcc: Face centered cubic
FAOR: Formic acid oxidation reaction
FC: Fuel cell
HER: Hydrogen evolution reaction
H_UPD_: Hydrogen underpotential deposition
MNC: Metal–nitrogen–carbon
OER: Oxygen evolution reaction
ORR: Oxygen reduction reaction
RHE: Reversible hydrogen electrode
ESI: Electronic supporting information

## Data availability

Data is available here: https://nano.ku.dk/english/research/theoretical-electrocatalysis/katladb/formic_acid_oxidation_reaction/.

## Author contributions

The manuscript was written through the contributions of all authors. All authors have given approval to the final version of the manuscript.

## Conflicts of interest

There are no conflicts to declare.

## Supplementary Material

SC-013-D2SC05160E-s001
