# Supplementary material for: Correlations between experiments and simulations for formic acid oxidation
Source: Chem Sci. 2022 Oct 26;13(45):13409–17. doi: 10.1039/d2sc05160e (PMC9682913; doi:10.1039/d2sc05160e)
Supplement: SC-013-D2SC05160E-s001 [file SC-013-D2SC05160E-s001.pdf]

Electronic Supplementary Material (ESI) for Chemical Science.
This journal is © The Royal Society of Chemistry 2022

Supporting Information

Correlations between experiments and simulations for formic acid oxidation

Alexander Bagger,[†,‡,a] Kim D. Jensen,[‡,a] Maryam Rashedi,[a,b] Rui Luo,[a,c] Jia Du,[d] Damin Zhang,[d] Inês J. Pereira,[a] María Escudero-Escribano,[a,e,f] Matthias Arenz,[a,d] and Jan Rossmeisl[a]

[a]University of Copenhagen, Department of Chemistry Universitetsparken 5, 2100 Kbh-Ø, Denmark

[b]College of Science, University of Tehran, Enghelab square, Tehran, Iran

[c]School of Environmental and Biological Engineering, Nanjing University of Science & Technology, Nanjing 210094, China

[d]University of Bern, Department of Chemistry, Biochemistry and Pharmaceutical Sciences, CH-3012 Bern, Switzerland

[e]Catalan Institute of Nanoscience and Nanotechnology (ICN2), CSIC, Barcelona Institute of Science and Technology, UAB Campus, 08193 Bellaterra, Barcelona, Spain

[f]ICREA, Pg. Lluís Companys 23, 08010 Barcelona, Spain

Formic acid electrooxidation literature

To establish reasonable context to our study we conducted a relative extensive literature study of what we gauge to be relevant papers concerning the formic acid oxidation reaction (FAOR) for direct formic acid fuel cell (DFAFCs) applications. The papers by *Kolb*[1] and *Adzic*[2] are prime examples of how in-depth model studies on extended surfaces can help researchers as the right questions. Cyclic voltammograms CVs of Pd(*hkl*) and Pt(*hkl*) have been compiled in Figure S1.

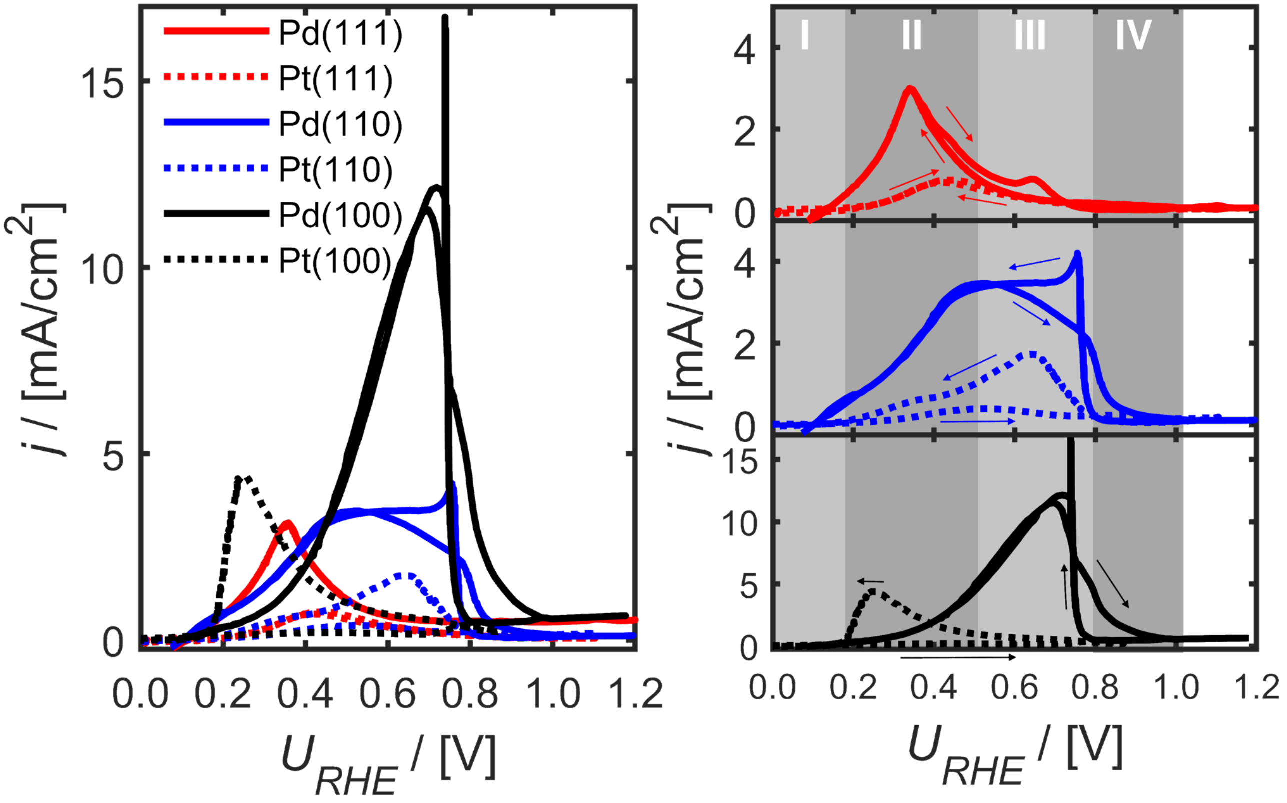

Figure S1. Literature study of CVs showing anodic and cathodic FAOR scans for basal planes Pt(*hkl*) and Pd(*hkl*). Pd data was taken from work by *Kolb* and coworkers[1] in 0.1 M H_2SO_4+0.2 M HCOOH. Pt data was from work by *Adzic et al.*[2] in 1 M $HClO_4$+0.26 M HCOOH; Note the designated potential regions; I: Ideal (low overpotential) FAOR region, overlaps with H_{UPD} region, II: FAOR region. III: CO oxidation region. IV: OH adsorption region.

Figure S1 allowed us establish the following:

- Pt(100) appears to exhibit the lowest onset potential at ~0.25 V_{RHE}, however as the catalyst shows complete inactivity on the cathodic sweeps, *i.e.* severe poisoning is suspected. In terms of FAOR onset order (FAOR peak position) it appears to follow that Pt(100)>Pd(111)>Pt(111)>Pd(110)>Pt(110)≈Pd(100).
- By considering the highest current as metric for activity[3] the order is then Pd(100)>Pt(100)> Pd(110)> Pd(111)>Pt(110)>Pt(111). *I.e.* it appears the more open fc structures shows better intrinsic activity.
- The surfaces showing least amount of difference between anodic and cathodic sweep are observed to have the following order:

  Pt(111)>Pd(111)>Pd(100)≈Pd(110)>Pt(110)>Pt(100)
- Similar Pd(*hkl*) work in 0.1 M $HClO_4$ by *Hoshi et al.*[3] shows similar trend in Pd activity albeit with higher maximum currents suggesting anion adsorption (*e.g.* (bi-)sulfates) may poison surfaces and thus influence FAOR activities. Recent work by *Koper and coworkers*[4] similarly highlight anions' importance during FAOR.

Figure S1 helped us to come to grip with the fact that more than one parameter is relevant to give insight to the FAOR performance and not just focus on maximum current. *E.g.* having an catalyst with extremely high current but high onset potential, such as Pd(100), essentially result in a DFAFC with very low cell voltages. From Figure 1 it appears that open (100)-like fcc surfaces allow for the highest currents, while closed (111)-like surfaces exhibit least differences between anodic- and cathodic sweeps, *i.e.* less prone to poisoning effects. It also appears as if Pt(100) allows for the lowest FAOR onset.[2] Unintuitive, it however appears that Pd(100) or Pd(911) is intrinsically more active.[1,3,5] We would like to note similar studies have been reported for Ir(*hkl*).

However, FAOR currents are extremely low (potentially due to irreversible surface oxidation), regardless we are confident that pure Ir is a poor FAOR catalyst.[6,7]

Expanding on Figure S1 it is important to note what is actually desirable traits for a FAOR catalyst when looking at its CV; *FAOR onset is desired to occur at low overpotential while exhibiting high currents (high intrinsic activity and limited poisoning). The anodic and cathodic sweeps should be comparable indicating little to no formation of poisoning species and/or catalyst restructuring. Finally, the electrode should be stable and active at relevant operational potentials, this is easier observed through chronoamperometric (CA) measurements*, see Figure S2.

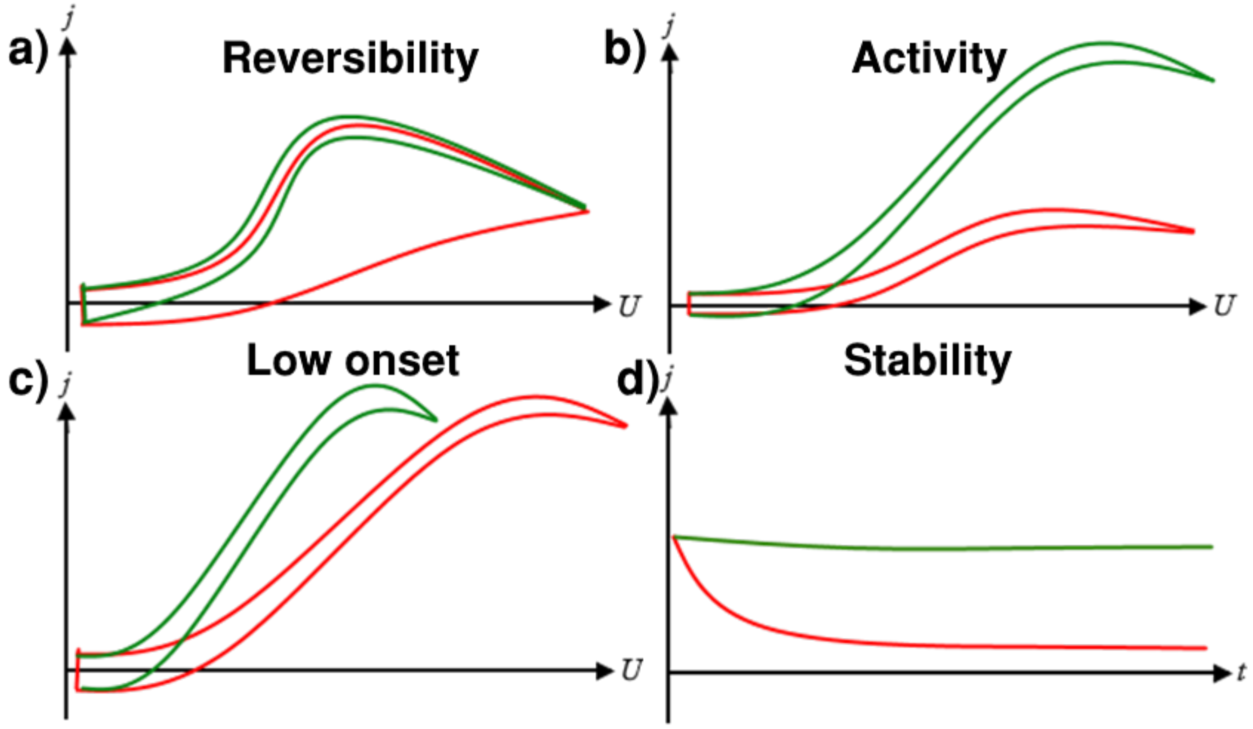

Figure S2. FAOR electrocatalytic performance scheme; (a) Anodic and cathodic sweep similar (no poisoning effect); (b) High activity *per* PGM mass (oxidation current); (c) Low overpotential and no need to strongly oxidizing potential for poison removal; (d) Stable performance.

The reason why current generally have been highlighted as the main performance metric is obvious given the main drawback of a DFAFC compared to a direct methanol fuel cell (DMFCs) lies in the energy density, where the overall $8e^-$ reaction in a DMFC follows:

$$\text{(DMFC)} \qquad CH_3OH + \frac{3}{2}O_2 \rightarrow 2H_2O + CO_2 \qquad , \Delta E_0\text{=1.2 eV} \qquad (1)$$

Conversely, the overall reaction in DFAFC only concerns $2e^-$:

$$\text{(DFAFC)} \qquad HCOOH + \frac{1}{2}O_2 \rightarrow H_2O + CO_2 \qquad , \Delta E_0\text{=0.72 eV} \qquad (2)$$

The DFAFC consist to two half reactions at the anode and the cathode, *i.e.* the formic acid oxidation reaction and the oxygen reduction reaction (ORR), respectively:

$$\text{(FAOR)} \; HCOOH \rightarrow CO_2 + 2H^+ + 2e^- \qquad , E_{0\rightarrow RHE}\text{=-0.23 eV} \qquad (3)$$

$$\text{(ORR)} \; \frac{1}{2}O_2 + 2H^+ + 2e^- \rightarrow H_2O \qquad , E_{0\rightarrow RHE}\text{=1.23 eV} \qquad (4)$$

From an electrocatalytic point-of-view optimizing FC catalysts for conversions with fewer reaction steps (charge transfers) is fundamentally easier, as the reaction intermediates' binding to the electrode surface typically will scale. Thus, optimizing the reaction intermediate binding for one species may inhibit the catalytic properties of the same reaction in a consequent reaction step *in lieu* of the *Sabatier* principle.[8] Due to FAORs industrially relevancy and the fundamental electrocatalytic insights one may derive from formic acid studies, it is a prime topic for mechanistic studies as illustrated by the impressive numbers of publications related to the subject. Most of these publications concerns catalysts based on platinum group metals (PGMs), typically Pd or Pt as these are the most active- and stable pure metal candidates for the catalytic reaction.[9,10] Rh can be considered similarly active,[11] but due its cost and scarcity[12] it is considered impractical for real world applications. In this work we have focused on approaches modifying commercially available Pt or Pd nanoparticles (NPs) and systematically compare these results with literature results and density functional theory (DFT) predictions. Our goal has

been to unify years of FAOR research using simple simulations and experiments elucidating appropriate design principles and fundamental limitations when developing catalyst.

From the preceding, it is clear that FAOR selectivity (between reaction and poisoning intermediates) is a fundamental aspect governing catalyst performance. Moreover, general FAOR trends can be extrapolated from Figure S1 and literature[13]: FAOR onset often seem to match hydrogen desorption potentials >0.2 V_{RHE}. It is in that aspect noted that Pd is reported to form Pd-hydride α- and β-phases below 0.2 V_{RHE}.[14]

Looking at selected FAOR data on nanocatalysts in literature[1,15] one could suspect that formic acid adsorption is limited by $^*OH$ and $^*H$ as FAOR activities are low in these regions and given the high FAOR activity in the 0.55-0.80 V_{RHE} region matches the potential at which neither H and OH is typically adsorbed on Pt(111) or Pd (111). Moreover, this is the potential region in which CO is oxidized *e.g.* CO oxidation occur at ~200 mV lower potentials on Pd(100) than its Pd(111) and Pd(110) counterparts. Perhaps Pd(100)'s activity can been ascribed to its ability to remove poisoning CO species at lower overpotential.[16,17] It is well-established that CO oxidation flows the trend (100)>(110)>(111) on both Pt[18,19] and Pd[16]. The question then arises*: Why, does Pd(111) or Pd(111) not exhibit high FAOR at potentials above their respective CO oxidation potentials?* Such question is strongly implied by Figure S1, and highlighted by the four different potential regions I-IV. Region I denotes the potential range in which FAOR theoretically should be able to occur, see Figure S1. Region II the earliest observed FAOR onset, note the anodic and cathodic sweeps are congruous with one another, suggesting one specific reaction pathway in which poisoning species are formed. Region III designate the region in which both CO oxidation and FAOR takes place, high activity in this potential region may be achieved through partly

oxidation of HCOOH to CO followed by CO oxidation toward CO_2. Its should be stressed, that any FAOR activity in region III is of no consequence in a real DFAFC as ORR overpotential will make any cell potential negligible and stack cost too high.[20] Finally, Region IV in Figure S1 designates a region in which FAOR is limited either due to formed site blocking species (other than *CO)[21] or the adsorption of other spectator species, such as *OH. We note that high activities in the III-IV potential region in Figure S1 are not relevant for DFAFC applications as significant ORR overpotentials are expected.[22] Moreover, at potentials above 1.1 V_{RHE} Pd and Pt is known to dissolve.[23,24]

Having the ability to detect adsorption onset is crucial to identify which adsorbates, spectator species and reaction intermediates (both warranted and unwarranted) are present during FAOR, is crucial as such information will identify the most likely reaction pathways and possible strategies for optimizing the FAOR catalyst design. Interestingly, FTIR studies by *Cuesta et al.* indicated that formate most likely is a reaction intermediate from FAOR on Pt[25] and Au[26]. However, it is not quite clear whether formate is an active intermediate or an poisoning intermediate blocking FAOR.[27] Similarly, *in situ* FTIR are not consistent over the literature in their interpretation and detection of CO[28,29] during FAOR.[30–33] This is a real issue; hardly any consistent *in situ* studies exist able to detect the presence of CO as a reaction poison exist. It does however appear from careful work on Pt(111) by *Arenz et al.*[34] that HCOOH forms CO at cathodic potentials which is removed above 0.6 V_{RHE} in line with our observations. Moreover, formate intermediate detection is proven using *in situ* FTIR techniques on NPs. However, as formate is both considered a necessity in the FAOR pathways and suspected as a poison standard FTIR studies may not be that useful in establishing FAOR insights. Rather, as most FAOR studies proposes reaction pathways that explains their electrochemical observations we decided

to do the same. Doing what other people already have done is essentially foolish, but using DFT to evaluate the relevant reaction steps energetics and consequent likelihood was hoped to give a unified explanation of what happens at the catalysts surface during FAOR. Unfortunately, FAOR is an extensive studied field and a cornucopia of reaction pathways have been considered in literature.[9,10,21,35–40] Hence, our principal contribution has been reducing the reaction space proposed in literature. We considered both reactions taking place during steady state FAOR and those potentially able to occur during cycling.

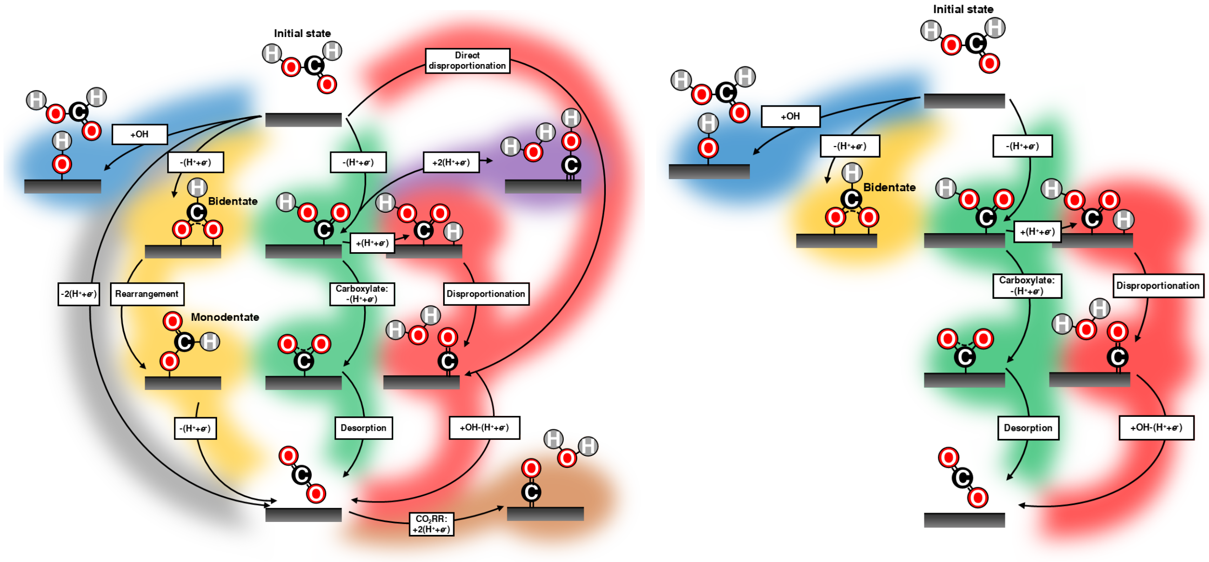

a) **b)**

Figure S3. Literature study highlighting all the conceived FAOR reaction pathways during potential cycling.[21] (a) Depicts all pathways indiscriminate of their likelihood proposed in literature. (b) Highlights the reduced number of pathways needed to understand low overpotential FAOR. Note historically FAOR has been split into the direct (*gray*)[35,36] and indirect FAOR (*green* and *yellow*)[35,37,38,41] FAOR pathway. It is worth noting that during potential cycling a multitude of possible reaction pathways and possible adsorption events are able to cloud any FAOR trend, *e.g.* from partial FAOR forming unwarranted CO_xH_y surface species (*Purple*),[9,10,39] site blockage due to hydroxide adsorption (*blue*) or formation of CO from

direct/indirect disproportionation (*red*)[40] has been proposed. Even CO_2 reduction reaction by cycling too cathodic (*brown*)[36] have been suggested in literature (reverse CO_2 reduction reaction). Note some of the reaction pathways like hydroxyl adsorption and CO-oxidation often takes place at relatively high potentials for Pt and Pd based catalysts. Moreover, while $^*CO$ and $^*CO_xH_y$ (and $^*OH$/$^*H$) are the most mentioned poisoned species mentioned in FAOR in literature, recent focus have shifted to formate itself (*yellow*), which in various arrangements may itself cause self-poisoning of the catalyst.[25,35,42,43]

From Figure S3 the all the relevant reaction pathways (and intermediates) have been established. As we *a priori* not know the true reaction route(s) and our identification of this/these pathways(s) are limited due to the fact that CO, formate, H and OH adsorption is difficult to detect with traditional FTIR techniques under *in situ* FAOR conditions[30–33]. Hence, herein we have opted for the simpler approach of testing catalysts with specific properties and compare these with widely studied reference systems before finally correlating these results with a broad self-consistent theoretical framework. Consequently, it is important to figure out which types of catalyst have been considered most relevant for the field. Given that our framework should encompass the results, obtained for the most studied catalysts in literature. In literature there has generally been four approaches to optimize FAOR performance (note other exists[44,45]):

1) Use intrinsically active materials such as Pt and Pd (and possible Rh[11]).
2) Design single-site catalyst; surround some active with inert Au,[46–48] Ag,[49] N/C[50] or other[51–54] as this should disallow the formation of CO during formic acid oxidation. Note, Rh and Ir single-sites have also been reported.[55–58]

3) Include ad-atoms on Pt or Pd catalyst as elements such as Cd,[59] Sn,[60] Sb,[61–63] Bi,[42,64–67] Pb,[39] Te,[68] and Tl[45,69] as these elements have been proposed to mitigate both CO and/or formate poisoning.
4) Alloy Pt or Pd, typically with Ru[70,71] as this has been shown to mitigate CO poisoning in DMFC systems. However, a range of other alloys systems bulk[49,72–83] and surface alloys[84] have also been proposed. We note most alloys exhibit similar FAOR performance regardless of Pt's or Pd's alloying elment.[85]

Tuning of catalyst shape and -morphologies and even active area have resulted in very conservative improvements in catalysts FAOR performances. A range of fundamental work on different (non-Pd, non-Pt, non-Rh) pure metal catalysts exist Ru,[86] Os,[87] Au.[26,88] However, currents magnitudes and/or onset potential was poor in all instances.

Several FAOR studies concerns the Pd-Au and Pt-Au systems. Recent work by *Zhang and coworkers* on Pt-Au NP single-site catalyst suggested some FAOR improvement using a novel, albeit not straightforward synthesis.[46] However, looking into the mass activity performance on many of these systems, we noted that all Pt-Au NPs systems' catalytic performances found in literature[89–93] are quite similar to one another; in-fact it seems to be true for all Pt_xM_{x-1}/Pd_xM_{x-1} alloys.[49,72,81–85,73–80] However, we wanted to see if previous results on Pt-Au systems arises from some unknown propensity towards forming Pt/Pd dual- or triple-sites during synthesis formation. One simple approach to test this is to form Pt_xM_{x-1} (M=Au, Ag, Hg and x>3) intermetallic. Fortunately work by *Arnau et al.*[94] have reported Hg_4Pt catalyst preparation by modifying Pt/C NPs and forming Pt-Hg. Our idea was to use this well-tested catalysts and see if we observe any mass activity improvement relative to the pure seed catalyst.

Other work by *Lim* and co-wokers[67] (among others) report Pt/C NP modification using Bi. Such model systems seems to exhibit very high activities and are consequently very interesting to us. Besides the work on various metal-nitrogen-carbon (MNC)and Pt/Pd alloyed catalyst it is worth mentioning that a range of fundamental works exist investigating the effects of electrolyte pH,[25,38,95,96] electrolyte composition,[97] HCOOH concentration/mass-transport limitations,[98,99] and temperature[42].

From the vast amount of literature concerning FAOR, establishing a robust theoretical framework explaining catalysts performing in all the potential regions, see Figure S1. Such a model should explain the limited activity of the most relevant pure metal catalysts, as well as relevant alloys and single-site systems (carbon-based or alloyed). We know from earlier work,[100] that catalytic properties changes significantly when considering carbon-based structures such as metal-nitrogen-carbon (MNC). Hence, our theory should consider elements relevant for both pure metal catalyst (see Figure S4a), bimetallic alloys and carbon-based motifs such as MNCs. The question then arises which metal and catalyst should we consider in our model to get a satisfactory model.

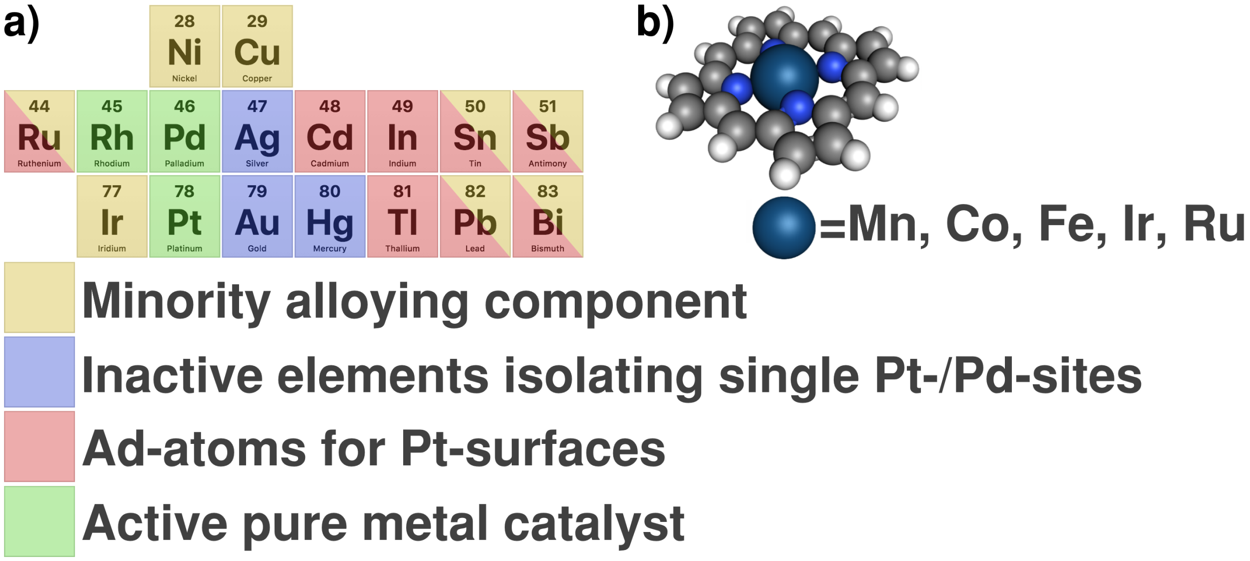

Figure S4. Elements relevant for screening FAOR catalysts. (a) Elements for metal based catalysts. Note; *Green* elements are the only active pure metal candidates. *Blue* elements are typically alloyed with *green* elements, in many instances in the hope of forming single-site catalysts. Alloys combining *green* and *yellow* elements are ripe in literature and are often proposed to increase either catalyst area or CO- or formate tolerance. Red elements are often reported as elements used to modify *green* elements by ad-layer/atoms. (b) Porphyrin MNC structures with relevant elements.

Besides the elements mentioned in Figure S4 ions of Cr, V, Co are known to oxidize HCOOH in homogenous catalysis.[101] From literature we also noted that very few model studies exist investigating other pure metal catalyst or alloys for FAOR, consequently we collected data on Ag, Cu and Ni as well as Pt_5M (M=Tb, Gd, La, Tm, and Ce) electrodes.[102]

Electrochemical experimental details

All electrochemical measurements were conducted in cleaned glassware in 0.1 M $HClO_4$ electrolytes (Milli-Q H_2O, 18.2 Ωcm, Merck, Suprapur® 30%). Rotating disk electrodes measurements relying on commercial Pt/C (TKK, 46.5 wt.% Pt, TEC10EA50E, *ca.* 5 mg_{cat} ink loading) and Pd/C (Aldrich, 407305-1G, 30 wt% Pd, *ca.* 5 mg_{cat} ink loading) catalysts using inks consisting of 2.5 mL 2-propanol (Aldrich, trace metal basis, 99.999%), 10 µL Nafion™ (Aldrich, aqueous suspension, 10 wt.%), 7.45 mL milli-Q H_2O.[103] Ink were mixed in the order: Catalyst, water, solvent and finally Nafion™ followed by 15 min sonication. 10 µL ink was dispersed on polished and cleaned glassy carbon stubs (HTW, $Ø \times h$=5×4mm) and set to dry under ambient conditions. Even surface distribution of ink was verified by visual inspection.[104] Mass activity was derived from total mass loading in the ink relative to the 10 µL loading put on the

electrodes. FAOR investigations were conducted in 0.1 M $HClO_4$ with 0.1 M HCOOH (Aldrich, ACS reagent, 98%) under rotation to avoid any possible mass-transport issues.[99] Reversible hydrogen electrode (RHE) potential and Ohmic-drop compensation was utilized.[105] Pt-Hg/C and Pd-Hg/C was prepared following earlier established methodology[94] on glassy carbon stubs pre-loaded with Pt/C or Pd/C ink and alloying in ~3mM HgO (Aldrich, trace metal basis, 99.999%) in 0.1 M $HClO_4$. Similarly, Pt-Bi/C preparation was based on the deposition procedure, see SI, of ~7 mM BiO_2 (Aldrich, trace metal basis, 99.999%) dissolved in 0.1 M $HClO_4$ as discussed by *Feliu et al*.[106]

Prior FAOR experiments reproducible CVs were obtained in pure HClO4 for the Pd/C, Pt/C, Pd-Hg/C, Pt-Hg/C and Pt-Bi/C catalyst, see figure 2a. Once we established reproducible Pt and Pd base CVs corresponding Pt-Hg, Pd-Hg and Pt-Bi CVs were obtained.

The Pt-Hg, Pd-Hg and Pt-Bi catalyst relied on deposition in a separate cell, and cathodic deposition of either Hg or Bi, see Figure S5.

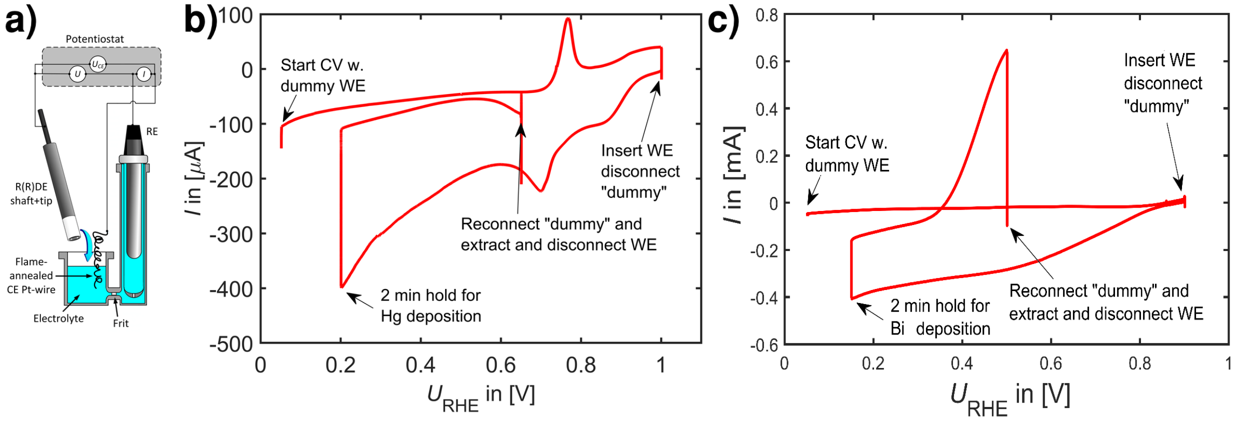

Figure S5. (a) Deposition cell used to alloy Pt and Pd with Hg and deposit Bi on Pt/C. (b) Hg alloying example on Pt (Pd looks similar). (c) Deposition of Bi ad-atoms on Pt NPs.

The deposition of Bi on Pt and the mercury alloying followed the procedure developed by others.[107]

We also tried depositing Pb following others work[39]. Unfortunately we found that Pb was unstable even at rather low potential <0.6 V_{RHE}, see Figure S6a. Similarly, we found that cycling Pt-Bi/C with Ohmic drop compensation stripped off Bi when going too high in potential. Hence, the Pt-Bi/C system was only considered stable in the 0.0-0.8 V_{RHE} range, see Figure S6b.

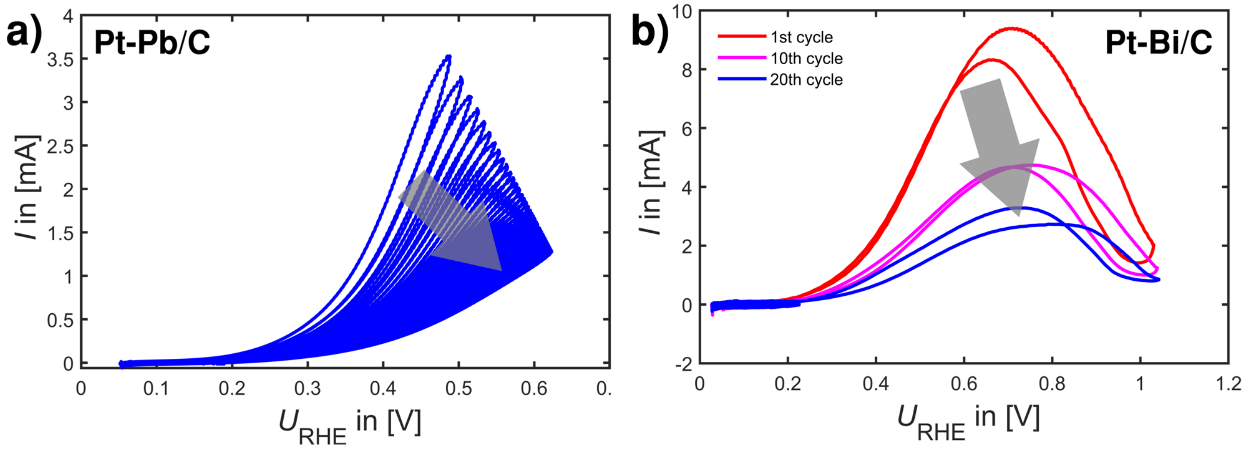

Figure S6. 20 first CVs at 50 mV/s, room temperature, 1600 rpm. (a) Deactivation of Pt-Pb/C catalyst, likely due Pb-oxidation and dissolution (b) Deactivation of Pt-Bi/C catalyst, likely due Bi-oxidation and dissolution.

Figure S6 highlights the instability of Pt-Pb/C and Pt-Bi/C systems and why these have not found use in industry for FAOR applications.

After deposition the electrochemical FAOR investigation relied on the following simple procedure: *i*) Electrodes were rinsed in milli-Q water and inserted into the electrochemical cell under potential control at potentials <0.25 V_{RHE}. *ii*) 200 CVs at 200 mV/s were obtained as a conditioning step, see example in Figure S7. *iii*) Electrochemical impedance spectroscopy (EIS)

0.55 V_{RHE} was obtained identifying the series resistance following earlier work. *iv*) Ohmic drop compensated (85%) CVs at 200, 100, 50, 20 and 10 mV/s were obtained at 1600 rpm, see Figure S8. All CVs were post corrected the remaining 15% of the Ohmic drop. *v*) Chronoamperometric measurements was collected these relied on potential steps at 0.05 V_{RHE} followed by 1.05 V_{RHE} for 30 s followed by 30 min hold at 0.55 V_{RHE} with 85% Ohmic drop compensation. The idea was to first reduce sample completely then oxidize and strip off all poisons and then conduct an extended CA measurements at a relevant potential. On occasion, a limited steps procedure was also used to highlight catalyst types propensity towards poisoning, see Figure 4.

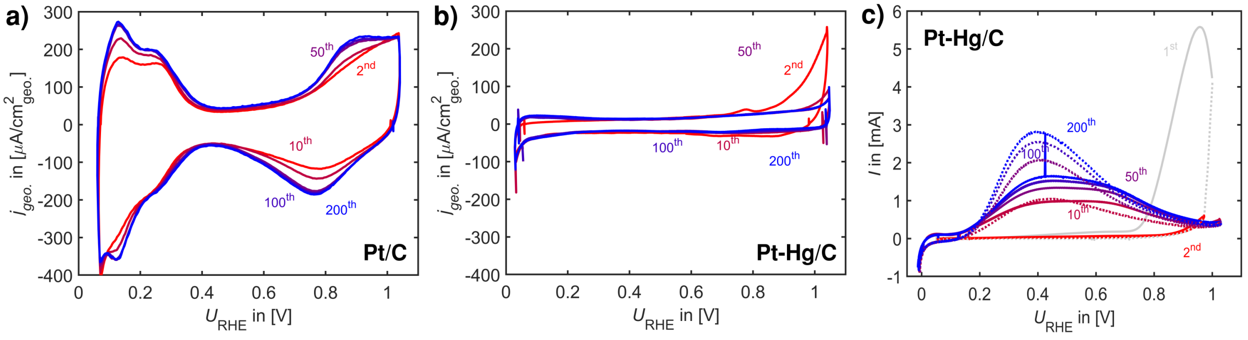

Figure S7. Conditioning step at 50 mV/s for 200 cycles in Ar-saturated electrolytes. (a) Pt/C in 0.1 M $HClO_4$. (b) Pt-Hg/ C in 0.1 M $HClO_4$. (c) Pt-Hg in 0.1 M $HClO_4$ with 0.1 M HCOOH. Note, that after 100 cycles CVs hardly changes.

Figure S7 reveals that *pseudo* stable CVs are obtainable after 200 cycles at 50 mV/s. However, in the interest of time and in order to limit time in which electrodes could experience some unforeseen poisoning event 200 mV/s was used as a conditioning step for the vast majority of our measurements.

CVs for Pt/C, Pd/C, Pt-Hg/C and Pd-Hg/C catalyst was conditioned and obtained in the potential limits from 0.00 to 1.05 V_{RHE}, whereas for Pt-Bi/C conditioning and CVs was limited to 0.0 to

0.8 V_{RHE}. Following the conditioning the aforementioned CVs were collected at varying can rates, see Figure S8.

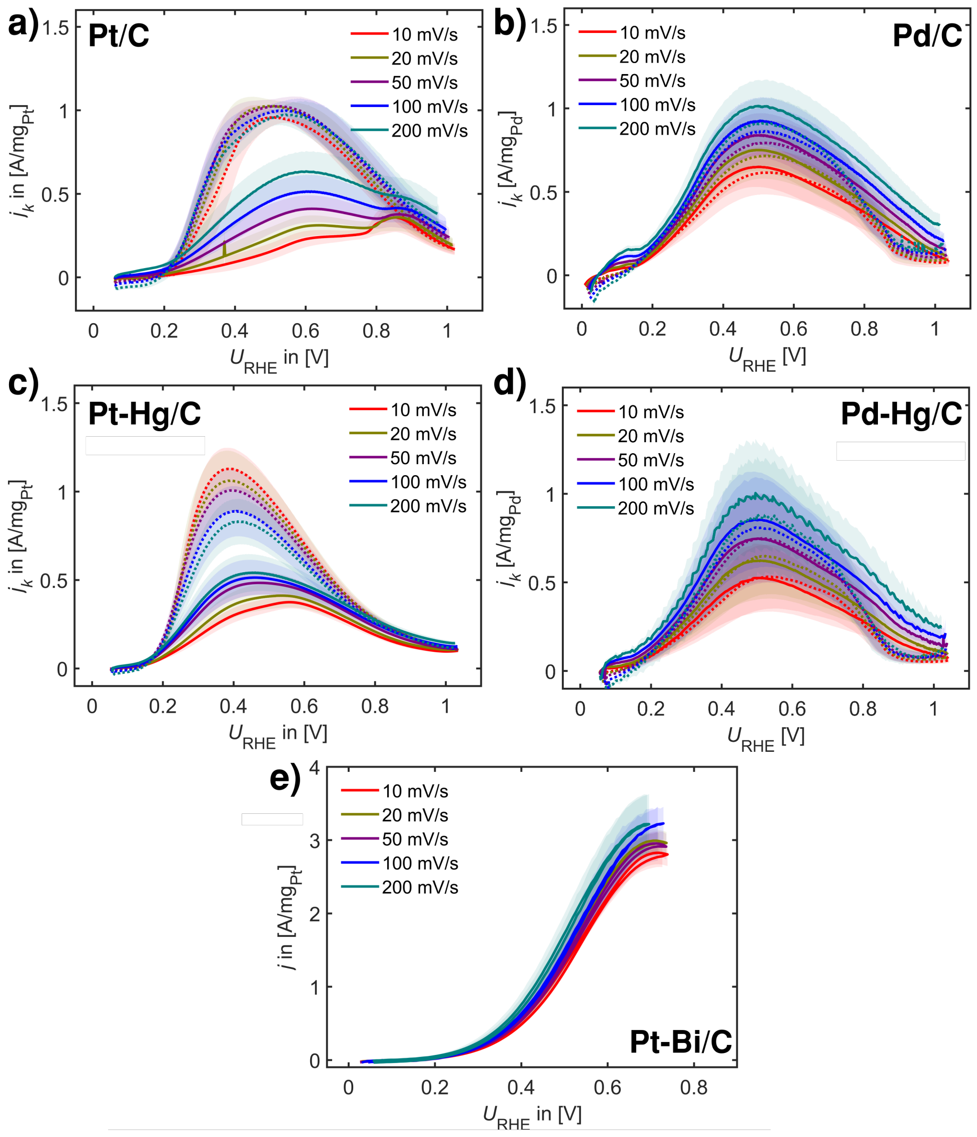

Figure S8. FAOR CVs at varying scan-speeds at taken at 1600 rpm in room-temperature Ar-saturated 0.1 M $HClO_4$ with 0.1 M HCOOH. (a) Pt/C NPs. (b) Pd/C NPs. (c) Pt-Hg/C NPs. (d) Pd-Hg/C NPs. (e) Pt-Bi/C NPs.

Figure 8 reveals that Pt/C, Pt-Hg/C and to some extend the Pd-Hg/C system is more sensitive to scan rate than say the Pd/C and Pt-Bi/C system.

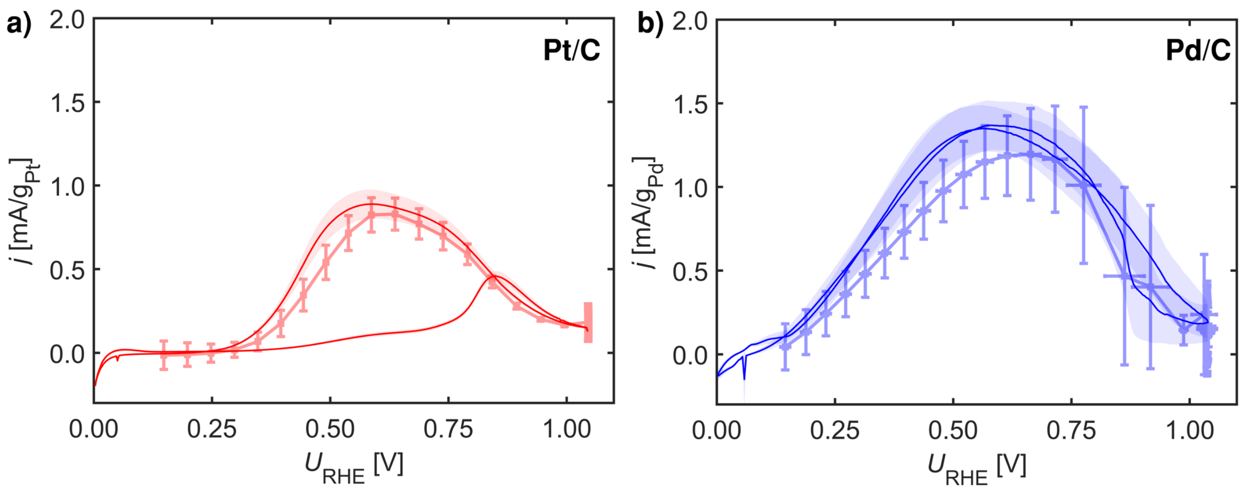

Figure S9. Pulsed voltammetry as that of Figure 4 utilizing 30 s pulses to justify this interpretation was insufficient as not enough *OOCH species to accumulate. (a) Pt/C. (b) Pd/C. Results are similar as those of Figure 4, suggesting OOCH does not accumulate noteworthy on Pd.

Additional electrochemical data

As we needed additional insight to pure metal catalyst for our theoretical framework we tested Ni, Ag and Cu wires as *ad hoc* test to elucidate whether CO- and H-binding were sufficient descriptors for FAOR activity, see Figure S10.

Due to one of our initial hypothesis, we hoped to observe CO-vibration absence for the Pt-Hg/C relative to the pure Pt/C system during *in situ Fourier*-transform infrared spectrometry (FTIR). Unfortunately, we were never able to observe CO-vibration changes in $HClO_4$ while varying the

potential. However, in flowing CO directly into the cell and conducting CO-stripping experiments revealed that if changes in CO-coverages were in-fact occurring these would not be observable in HCOOH containing electrolyte, see Figure S10.

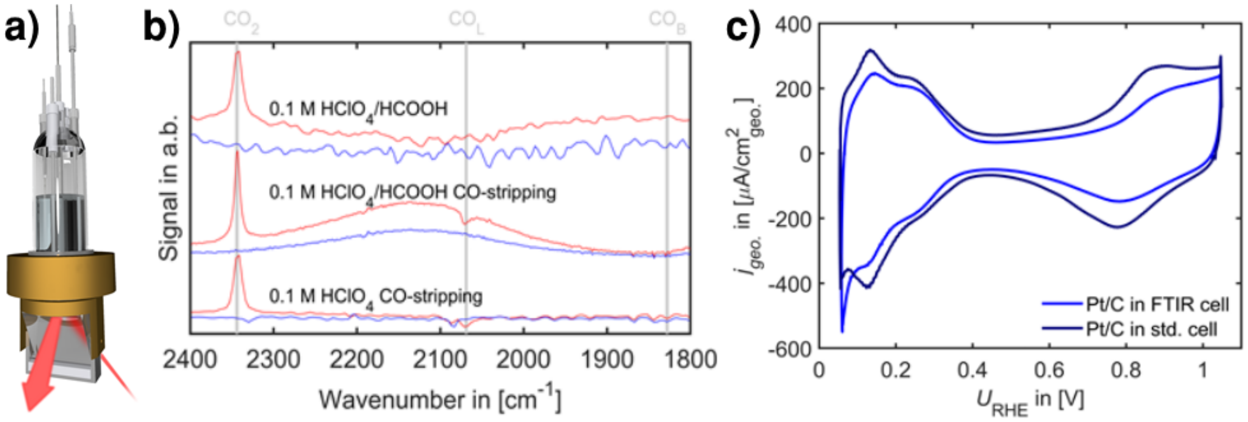

Figure S10. (a) Schematic of FTIR cell used. (b) *In situ* FTIR spectra of Pt/C NPs *blue*-background taken at 0.05 V_{RHE} and *red*-spectra at 1.05 V_{RHE}. (c) Comparison of CVs in a standard three-electrode cell and the developed FTIR cell.

From our disappointing FTIR results and the somewhat inconclusive literature study on the subject we came to the conclusion that standard FTIR techniques are incompatible with what we expected to observe. Additionally, we noted from our extensive literature study that tuning of Pt-based catalyst have been seldom explored for the FAOR. This was done in an effort also to track CO-stretching for these alloys.

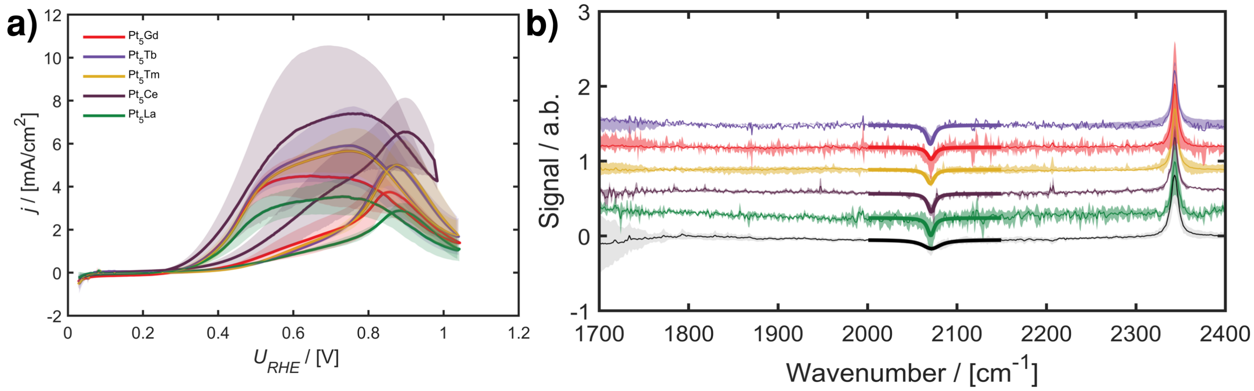

Figure S11. (a) CVs of Pt$_5$*M* extended surface alloys at 10 mV/s at room-temperature and 1600 rpm in Ar-saturated 0.1 M $HClO_4$ with 0.1 M HCOOH. (b) *In situ* FTIR CO-stripping of these alloys in pure 0.1 M $HClO_4$.

The most surprising aspect of the FTIR study of Figure S11 was that contrary to our expectation the CO-stripping peak did not seem to shift significantly with the lanthanide contraction[102] and consequently the CO-binding strength[109]. In regards to the FAOR data the Pt$_5$*M* data strongly suggests that binding of CO is unlikely the limiting factor for most of the observed FAOR performances presented in within the field, and consequently any theoretical model should take this into account.

From our realization, that disproportionation from *H and HCOOH (or *CHOO) towards *CO and H_2O was a real issue in Pt- (and perhaps) Pd-based catalysts, we wondered if we by shifting the HCOOH adsorption beyond the pKa of 3.75 could minimize disproportionation. Hence, CVs in pH 4.2 in formic acid and acetate was attempted, see Figure S12.

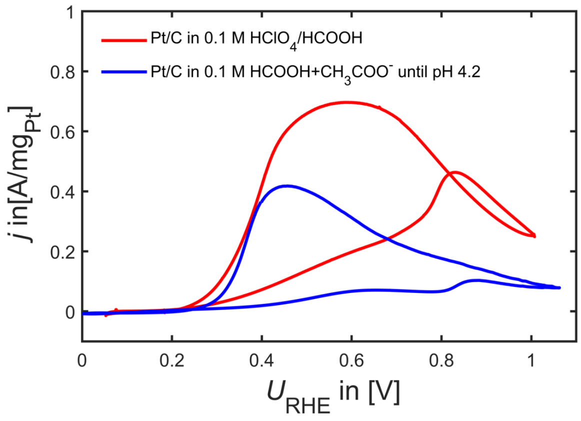

Figure S12. CVs of Pt/C in Ar-saturated 0.1 M HCOOH in 0.1M $HClO_4$ (*red*) or with CH_3COO^- (*blue*), pH measured 4.2, taken at 50 mV/s at room-temperature at 1600 rpm.

Figure S12 suggested that tuning pH was unlikely to influence FAOR activity in any meaningful way. This was done by going to pH's above formic acid's pKa of 3.75. Although a change in activity is visible the onset hardly varies. Any effects could simply be due to acetate adsorption. Or in other words, changing pH appears not to have an effect on the hysteresis, suggesting the poisoning seen as hysteresis is independent on HCOOH's pKa, *i.e.* facilitated by something that is not HCOOH.

It was generally noted, that getting reproducible results of the FAOR performance was very difficult *in lieu* of the NP ink system. It seemed that the catalyst surfaces were very dependent on the cleanliness and age of the electrodes on which ink was deposited. This very real dependence on advantageous adsorption and its effect on FAOR have been highlighted by adding HCl into our $HClO_4$/HCOOH electrolyte during CVs, see Figure S13.

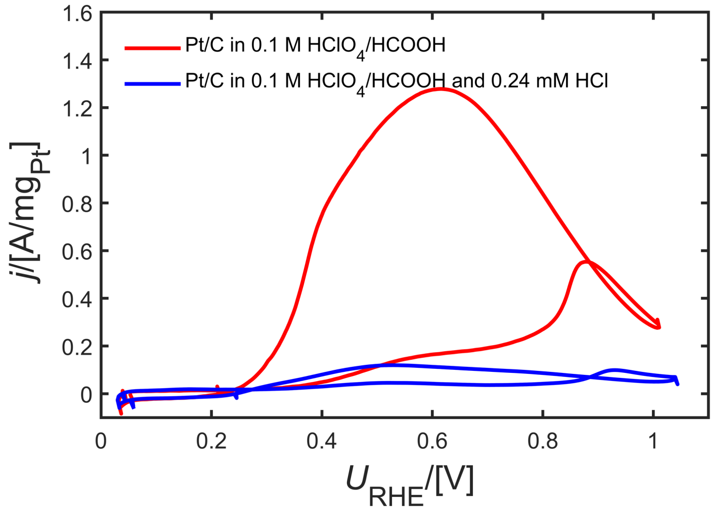

Figure S13. Base CV of Pt/C in Ar-saturated 0.1 M HCOOH and 0.1 M $HClO_4$ taken at 50 mV/s at room-temperature at 1600 rpm (*red*) into which a small amount 0.1M $HClO_4$ with 0.1 M HCl was added (*blue*).

The devastating effect HCl has on FAOR highlights how certain adsorbates can significantly lower FAOR activities.

Density Functional Theory (DFT) Calculations Details

All calculations were performed using Density Functional Theory (DFT), using the programs ASE version 3.19.0,[110] and GPAW version 19.8.1.[111] Calculations were done at the Generalized Gradient Approximation (GGA) level of theory, using the grid mode, with the BEEF-VdW functional.[112] All calculations are done in vacuum. We utilize a *k*-point sampling appropriate for the specific structure and a vacuum of minimum 10 Å. All the structures are relaxed to a force below 0.05 eV /Å. As model structure, we use the (111)-fcc facet to represent the metal catalyst, which is a fair choice when analysis is carried out on eV scale (although we note in Figure S1

that (100)-facets may be more active than (111)). As MNC model structure, we use our previous model site with four coordinated nitrogen atoms to the metal in a graphene sheet. All structures (metals, MNCs, $Pt_1Au(111)$, $PtHg_4$ and $PdHg_4$) are accessible through our online databases at https://chem.ku.dk/research_sections/nanochem/theoretical-electrocatalysis/

Table S1: Binding energy data, using H_2 and CO_2 as references.

Structure Name	H [eV]	COOH [eV]	OOCH [eV]
metal: Au	0.40	0.79	0.57
metal: Ag	0.43	0.90	-0.02
metal: Cu	0.02	0.54	-0.35
metal: Ni	-0.31	0.13	-0.55
metal: Pd	-0.31	0.05	0.01
metal: Pt	-0.23	-0.17	0.07
metal: Rh	-0.26	-0.05	-0.45
metal: Cd	0.73	0.92	-0.23
metal: Fe	-0.46	-0.38	-1.22
metal: In	0.85	0.68	-0.53
metal: Ir	-0.20	-0.12	-0.42
metal: Pb	0.91	0.90	-0.21
metal: Sn	0.83	0.65	-0.29
metal: Tl	0.87	0.86	-0.38
MNC: Fe	0.30	0.24	0.08
MNC: Co	0.16	0.14	0.29
MNC: Ir	-0.33	-0.22	0.52
MNC: Mn	0.44	0.41	-0.10
MNC: Rh	-0.13	-0.01	0.45
MNC: Ru	-0.54	-0.57	-0.30
PdHg4	0.36	0.26	0.18
PtHg4	-0.20	-0.19	0.06
Pt1Au(111)	-0.14	-0.05	0.24

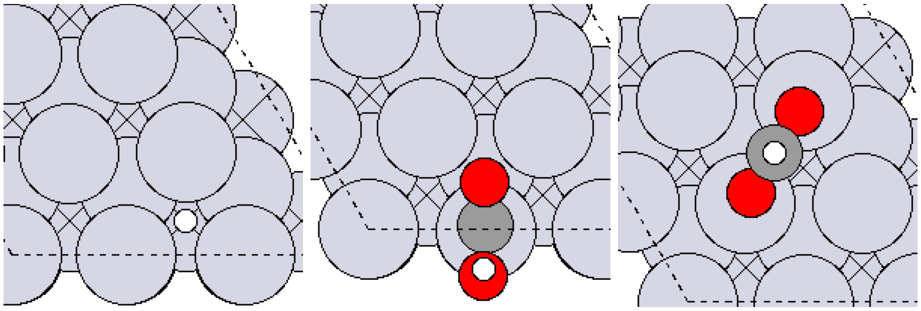

Figure S14. Binding energy motifs of *H, *COOH and *OOCH on Pt(111)

References

(1) Baldauf, M.; Kolb, D. M. Formic Acid Oxidation on Ultrathin Pd Films on Au(Hkl) and Pt(Hkl) Electrodes. *J. Phys. Chem.* **1996**, *100* (27), 11375–11381. https://doi.org/10.1021/jp952859m.

(2) Adžić, R. R.; Tripković, A. V.; O'Grady, W. E. Structural Effects in Electrocatalysis. *Nature* **1982**, *296* (5853), 137–138. https://doi.org/10.1038/296137a0.

(3) Hoshi, N.; Kida, K.; Nakamura, M.; Nakada, M.; Osada, K. Structural Effects of Electrochemical Oxidation of Formic Acid on Single Crystal Electrodes of Palladium. *J. Phys. Chem. B* **2006**, *110* (25), 12480–12484. https://doi.org/10.1021/jp0608372.

(4) Chen, X.; Granda-Marulanda, L. P.; McCrum, I. T.; Koper, M. T. M. How Palladium Inhibits CO Poisoning during Electrocatalytic Formic Acid Oxidation and Carbon Dioxide Reduction. *Nat. Commun.* **2022**, *13* (1), 1–11. https://doi.org/10.1038/s41467-021-27793-5.

(5) Grozovski, V.; Climent, V.; Herrero, E.; Feliu, J. M. Intrinsic Activity and Poisoning Rate for HCOOH Oxidation at Pt(100) and Vicinal Surfaces Containing Monoatomic (111) Steps. *ChemPhysChem* **2009**, *10* (11), 1922–1926. https://doi.org/10.1002/cphc.200900261.

(6) Gómez, R.; Weaver, M. J. Electrochemical Infrared Studies of Monocrystalline Iridium Surfaces Part I: Electrooxidation of Formic Acid and Methanol. *J. Electroanal. Chem.* **1997**, *435* (1–2), 205–215. https://doi.org/10.1016/S0022-0728(97)00304-5.

(7) Motoo, S.; Furuya, N. Electrochemistry of Iridium Single Crystal Surfaces. *J. Electroanal. Chem. Interfacial Electrochem.* **1986**, *197* (1–2), 209–218. https://doi.org/10.1016/0022-0728(86)80150-4.

(8) Nitopi, S.; Bertheussen, E.; Scott, S. B.; Liu, X.; Engstfeld, A. K.; Horch, S.; Seger, B.; Stephens, I. E. L.; Chan, K.; Hahn, C.; Nørskov, J. K.; Jaramillo, T. F.; Chorkendorff, I. Progress and Perspectives of Electrochemical CO2 Reduction on Copper in Aqueous Electrolyte. *Chem. Rev.* **2019**, *119* (12), 7610–7672. https://doi.org/10.1021/acs.chemrev.8b00705.

(9) Capon, A.; Parsons, R. The Oxidation of Formic Acid at Noble Metal Electrodes Part 4. Platinum + Palladium Alloys. *J. Electroanal. Chem.* **1975**, *65* (1), 285–305. https://doi.org/10.1016/0368-1874(75)85124-0.

(10) Capon, A.; Parsons, R. The Oxidation of Formic Acid at Noble Metal Electrodes Part III. Intermediates and Mechanism on Platinum Electrodes. *J. Electroanal. Chem.* **1973**, *45* (2), 205–231. https://doi.org/10.1016/S0022-0728(73)80158-5.

(11) Ortiz, R.; Márquez, O. P.; Márquez, J.; Gutiérrez, C. Spectroelectrochemical Evaluation of Rh Microparticles as Electrocatalyst for Carbon Monoxide and Formic Acid Oxidation. *Port. Electrochim. Acta* **2006**, *24* (1), 105–116. https://doi.org/10.4152/pea.200601105.

(12) Vesborg, P. C. K.; Jaramillo, T. F. Addressing the Terawatt Challenge: Scalability in the Supply of Chemical Elements for Renewable Energy. *Rsc Adv.* **2012**, *2* (21), 7933–7947. https://doi.org/10.1039/c2ra20839c.

(13) Petrii, O. A. The Progress in Understanding the Mechanisms of Methanol and Formic Acid Electrooxidation on Platinum Group Metals (a Review). *Russ. J. Electrochem.* **2019**, *55* (1), 1–33. https://doi.org/10.1134/S1023193519010129.

(14) Rose, A.; Maniguet, S.; Mathew, R. J.; Slater, C.; Yao, J.; Russell, A. E. Hydride Phase Formation in Carbon Supported Palladium Nanoparticle Electrodes Investigated Using in Situ EXAFS and XRD. *Phys. Chem. Chem. Phys.* **2003**, *5* (15), 3220–3225. https://doi.org/10.1039/b302956e.

(15) Oezaslan, M.; Heggen, M.; Strasser, P. Size-Dependent Morphology of Dealloyed Bimetallic Catalysts: Linking the Nano to the Macro Scale. *J. Am. Chem. Soc.* **2012**, *134* (1), 514–524. https://doi.org/10.1021/ja20881621.

(16) Hara, M.; Linke, U.; Wandlowski, T. Preparation and Electrochemical Characterization of Palladium Single Crystal Electrodes in 0.1 M H2SO4 and HClO4. Part I. Low-Index Phases. *Electrochim. Acta* **2007**, *52* (18), 5733–5748. https://doi.org/10.1016/j.electacta.2006.11.048.

(17) Bezerra, C. W. B.; Zhang, L.; Lee, K.; Liu, H.; Marques, A. L. B.; Marques, E. P.; Wang,

H.; Zhang, J. A Review of Fe-N/C and Co-N/C Catalysts for the Oxygen Reduction Reaction. *Electrochim. Acta* **2008**, *53* (15), 4937–4951. https://doi.org/10.1016/j.electacta.2008.02.012.

(18) Cuesta, A.; Couto, A.; Rincón, A.; Pérez, M. C.; López-Cudero, A.; Gutiérrez, C. Potential Dependence of the Saturation CO Coverage of Pt Electrodes: The Origin of the Pre-Peak in CO-Stripping Voltammograms. Part 3: Pt(Poly). *J. Electroanal. Chem.* **2006**, *586* (2), 184–195. https://doi.org/10.1016/j.jelechem.2005.10.006.

(19) López-Cudero, A.; Cuesta, Á.; Gutiérrez, C. Potential Dependence of the Saturation CO Coverage of Pt Electrodes: The Origin of the Pre-Peak in CO-Stripping Voltammograms. Part 2: Pt(100). *J. Electroanal. Chem.* **2006**, *586* (2), 204–216. https://doi.org/10.1016/j.jelechem.2005.10.003.

(20) Gasteiger, H. A.; Garche, J. Fuel Cells. In *Handbook of Heterogeneous Catalysis*; 2008.

(21) Sun, S. G.; Clavilier, J.; Bewick, A. The Mechanism of Electrocatalytic Oxidation of Formic Acid on Pt (100) and Pt (111) in Sulphuric Acid Solution: An Emirs Study. *J. Electroanal. Chem.* **1988**, *240* (1–2), 147–159. https://doi.org/10.1016/0022-0728(88)80319-X.

(22) Nørskov, J. K.; Rossmeisl, J.; Logadottir, A.; Lindqvist, L.; Kitchin, J. R.; Bligaard, T.; Jónsson, H. Origin of the Overpotential for Oxygen Reduction at a Fuel-Cell Cathode. *J. Phys. Chem. B* **2004**, *108* (46), 17886–17892. https://doi.org/10.1021/jp047349j.

(23) Pizzutilo, E.; Geiger, S.; Freakley, S. J.; Mingers, A.; Cherevko, S.; Hutchings, G. J.; Mayrhofer, K. J. J. Palladium Electrodissolution from Model Surfaces and Nanoparticles.

Electrochim. Acta **2017**, *229*, 467–477. https://doi.org/10.1016/j.electacta.2017.01.127.

(24) Cherevko, S.; Kulyk, N.; Mayrhofer, K. J. J. Durability of Platinum-Based Fuel Cell Electrocatalysts: Dissolution of Bulk and Nanoscale Platinum. *Nano Energy* **2016**, *29*, 275–298. https://doi.org/10.1016/j.nanoen.2016.03.005.

(25) Joo, J.; Uchida, T.; Cuesta, A.; Koper, M. T. M.; Osawa, M. The Effect of PH on the Electrocatalytic Oxidation of Formic Acid/Formate on Platinum: A Mechanistic Study by Surface-Enhanced Infrared Spectroscopy Coupled with Cyclic Voltammetry. *Electrochim. Acta* **2014**, *129*, 127–136. https://doi.org/10.1016/j.electacta.2014.02.040.

(26) Cuesta, A.; Cabello, G.; Hartl, F. W.; Escudero-Escribano, M.; Vaz-Domínguez, C.; Kibler, L. A.; Osawa, M.; Gutiérrez, C. Electrooxidation of Formic Acid on Gold: An ATR-SEIRAS Study of the Role of Adsorbed Formate. *Catal. Today* **2013**, *202* (1), 79–86. https://doi.org/10.1016/j.cattod.2012.04.022.

(27) Feliu, J. M.; Herrero, E. Formic Acid Oxidation. *Handb. Fuel Cells* **2010**. https://doi.org/10.1002/9780470974001.f206048.

(28) Figueiredo, M. C.; Hiltrop, D.; Sundararaman, R.; Schwarz, K. A.; Koper, M. T. M. Absence of Diffuse Double Layer Effect on the Vibrational Properties and Oxidation of Chemisorbed Carbon Monoxide on a Pt(111) Electrode. *Electrochim. Acta* **2018**, *281* (111), 127–132. https://doi.org/10.1016/j.electacta.2018.05.152.

(29) Stamenkovié, V.; Arenz, M.; Ross, P. N.; Markovié, N. M. Temperature-Induced Deposition Method for Anchoring Metallic Nanoparticles onto Reflective Substrates for in Situ Electrochemical Infrared Spectroscopy. *J. Phys. Chem. B* **2004**, *108* (46), 17915–

17920. https://doi.org/10.1021/jp0467301.

(30) Chang, S.-C.; Ho, Y.; Weaver, M. J. Applications of Real-Time Infrared Spectroscopy to Electrocatalysis at Bimetallic Surfaces. *Surf. Sci.* **1992**, *265* (1–3), 81–94. https://doi.org/10.1016/0039-6028(92)90489-s.

(31) Chang, S. C.; Leung, L. W. H.; Weaver, M. J. Metal Crystallinity Effects in Electrocatalysis as Probed by Real-Time FTIR Spectroscopy: Electrooxidation of Formic Acid, Methanol, and Ethanol on Ordered Low-Index Platinum Surfaces. *J. Phys. Chem.* **1990**, *94* (15), 6013–6021. https://doi.org/10.1021/j100378a072.

(32) Antoniassi, R. M.; Erikson, H.; Solla-Gullón, J.; Torresi, R. M.; Feliu, J. M. Formic Acid Electrooxidation on Small, {1 0 0} Structured, and Pd Decorated Carbon-Supported Pt Nanoparticles. *J. Catal.* **2021**, *400*, 140–147. https://doi.org/10.1016/j.jcat.2021.05.026.

(33) Iwasita, T.; Xia, X.; Herrero, E.; Liess, H. D. Early Stages during the Oxidation of HCOOH on Single-Crystal Pt Electrodes as Characterized by Infrared Spectroscopy. *Langmuir* **1996**, *12* (17), 4260–4265. https://doi.org/10.1021/la960264s.

(34) Arenz, M.; Stamenkovic, V.; Schmidt, T. J.; Wandelt, K.; Ross, P. N.; Markovic, N. M. The Electro-Oxidation of Formic Acid on Pt–Pd Single Crystal Bimetallic Surfaces. *Phys. Chem. Chem. Phys.* **2003**, *5* (19), 4242–4251. https://doi.org/10.1039/B306307K.

(35) Gao, W.; Keith, J. A.; Anton, J.; Jacob, T. Theoretical Elucidation of the Competitive Electro-Oxidation Mechanisms of Formic Acid on Pt(111). *J. Am. Chem. Soc.* **2010**, *132* (51), 18377–18385. https://doi.org/10.1021/ja1083317.

(36) Rice, C.; Ha, S.; Masel, R. I.; Wieckowski, A. Catalysts for Direct Formic Acid Fuel Cells. *J. Power Sources* **2003**, *115* (2), 229–235. https://doi.org/10.1016/S0378-7753(03)00026-0.

(37) Rees, N. V.; Compton, R. G. Sustainable Energy: A Review of Formic Acid Electrochemical Fuel Cells. *J. Solid State Electrochem.* **2011**, *15* (10), 2095–2100. https://doi.org/10.1007/s10008-011-1398-4.

(38) Ferre-Vilaplana, A.; Perales-Rondón, J. V.; Buso-Rogero, C.; Feliu, J. M.; Herrero, E. Formic Acid Oxidation on Platinum Electrodes: A Detailed Mechanism Supported by Experiments and Calculations on Well-Defined Surfaces. *J. Mater. Chem. A* **2017**, *5* (41), 21773–21784. https://doi.org/10.1039/c7ta07116g.

(39) Xia, X. H.; Iwasita, T. Influence of Underpotential Deposited Lead upon the Oxidation of HCOOH in HClO4 at Platinum Electrodes. *J. Electrochem. Soc.* **1993**, *140* (9), 2559–2565. https://doi.org/10.1149/1.2220862.

(40) Tripković, A.; Popović, K.; Adžić, R. Structural Effects in Electrocatalysis : Oxidation of Formic Acid on Single Crystal Platinum Electrodes and Evidence for Oscillatory Behaviour. *J. Chim. Phys.* **1991**, *88* (January), 1635–1647. https://doi.org/10.1051/jcp/1991881635.

(41) Neurock, M.; Janik, M.; Wieckowski, A. A First Principles Comparison of the Mechanism and Site Requirements for the Electrocatalytic Oxidation of Methanol and Formic Acid over Pt. *Faraday Discuss.* **2009**, *140*, 363–378. https://doi.org/10.1039/B804591G.

(42) Ferre-Vilaplana, A.; Perales-Rondón, J. V.; Feliu, J. M.; Herrero, E. Understanding the

Effect of the Adatoms in the Formic Acid Oxidation Mechanism on Pt(111) Electrodes. *ACS Catal.* **2015**, *5* (2), 645–654. https://doi.org/10.1021/cs501729j.

(43) Gennero de Chialvo, M. R.; Luque, G. C.; Chialvo, A. C. Formic Acid Electrooxidation on Platinum, Resolution of the Kinetic Mechanism in Steady State and Evaluation of the Kinetic Constants. *ChemistrySelect* **2018**, *3* (34), 9768–9772. https://doi.org/10.1002/slct.201801725.

(44) Lee, J.; Yoo, J. K.; Lee, H.; Kim, S. H.; Sohn, Y.; Rhee, C. K. Formic Acid Oxidation on Pt Deposit Model Catalysts on Au: Single-Layered Pt Deposits, Plateau-Type Pt Deposits, and Conical Pt Deposits. *Electrochim. Acta* **2019**, *310*, 38–44. https://doi.org/10.1016/j.electacta.2019.04.107.

(45) Busó-Rogero, C.; Perales-Rondón, J. V.; Farias, M. J. S.; Vidal-Iglesias, F. J.; Solla-Gullon, J.; Herrero, E.; Feliu, J. M. Formic Acid Electrooxidation on Thallium-Decorated Shape-Controlled Platinum Nanoparticles: An Improvement in Electrocatalytic Activity. *Phys. Chem. Chem. Phys.* **2014**, *16* (27), 13616–13624. https://doi.org/10.1039/c4cp00304g.

(46) Duchesne, P. N.; Li, Z. Y.; Deming, C. P.; Fung, V.; Zhao, X.; Yuan, J.; Regier, T.; Aldalbahi, A.; Almarhoon, Z.; Chen, S.; Jiang, D. en; Zheng, N.; Zhang, P. Golden Single-Atomic-Site Platinum Electrocatalysts. *Nat. Mater.* **2018**, *17* (11), 1033–1039. https://doi.org/10.1038/s41563-018-0167-5.

(47) Kristian, N.; Yan, Y.; Wang, X. Highly Efficient Submonolayer Pt-Decorated Au Nano-Catalysts for Formic Acid Oxidation. *Chem. Commun.* **2008**, *1* (3), 353–355.

https://doi.org/10.1039/b714230g.

(48) Wang, R.; Liu, J.; Liu, P.; Bi, X.; Yan, X.; Wang, W.; Ge, X.; Chen, M.; Ding, Y. Dispersing Pt Atoms onto Nanoporous Gold for High Performance Direct Formic Acid Fuel Cells. *Chem. Sci.* **2014**, *5* (1), 403–409. https://doi.org/10.1039/c3sc52792a.

(49) Liu, D.; Xie, M.; Wang, C.; Liao, L.; Qiu, L.; Ma, J.; Huang, H.; Long, R.; Jiang, J.; Xiong, Y. Pd-Ag Alloy Hollow Nanostructures with Interatomic Charge Polarization for Enhanced Electrocatalytic Formic Acid Oxidation. *Nano Res.* **2016**, *9* (6), 1590–1599. https://doi.org/10.1007/s12274-016-1053-6.

(50) Bulushev, D. A.; Zacharska, M.; Shlyakhova, E. V.; Chuvilin, A. L.; Guo, Y.; Beloshapkin, S.; Okotrub, A. V.; Bulusheva, L. G. Single Isolated Pd2+ Cations Supported on N-Doped Carbon as Active Sites for Hydrogen Production from Formic Acid Decomposition. *ACS Catal.* **2016**, *6* (2), 681–691. https://doi.org/10.1021/acscatal.5b02381.

(51) Yang, S.; Chung, D. Y.; Tak, Y. J.; Kim, J.; Han, H.; Yu, J. S.; Soon, A.; Sung, Y. E.; Lee, H. Electronic Structure Modification of Platinum on Titanium Nitride Resulting in Enhanced Catalytic Activity and Durability for Oxygen Reduction and Formic Acid Oxidation. *Appl. Catal. B Environ.* **2015**, *174–175*, 35–42. https://doi.org/10.1016/j.apcatb.2015.02.033.

(52) El-Nagar, G. A.; Hassan, M. A.; Lauermann, I.; Roth, C. Efficient Direct Formic Acid Fuel Cells (DFAFCs) Anode Derived from Seafood Waste: Migration Mechanism. *Sci. Rep.* **2017**, *7* (1), 1–11. https://doi.org/10.1038/s41598-017-17978-8.

(53) Zhang, R.; Peng, M.; Ling, L.; Wang, B. PdIn Intermetallic Material with Isolated Single-Atom Pd Sites – A Promising Catalyst for Direct Formic Acid Fuel Cell. *Chem. Eng. Sci.* **2019**, *199*, 64–78. https://doi.org/10.1016/j.ces.2019.01.018.

(54) Shen, T.; Lu, Y.; Gong, M.; Zhao, T.; Hu, Y.; Wang, D. Optimizing Formic Acid Electro-Oxidation Performance by Restricting the Continuous Pd Sites in Pd-Sn Nanocatalysts. *ACS Sustain. Chem. Eng.* **2020**, *8* (32), 12239–12247. https://doi.org/10.1021/acssuschemeng.0c03881.

(55) Xiong, Y.; Dong, J.; Huang, Z. Q.; Xin, P.; Chen, W.; Wang, Y.; Li, Z.; Jin, Z.; Xing, W.; Zhuang, Z.; Ye, J.; Wei, X.; Cao, R.; Gu, L.; Sun, S.; Zhuang, L.; Chen, X.; Yang, H.; Chen, C.; Peng, Q.; Chang, C. R.; Wang, D.; Li, Y. Single-Atom Rh/N-Doped Carbon Electrocatalyst for Formic Acid Oxidation. *Nat. Nanotechnol.* **2020**, *15* (5), 390–397. https://doi.org/10.1038/s41565-020-0665-x.

(56) Shen, W.; Ge, L.; Sun, Y.; Liao, F.; Xu, L.; Dang, Q.; Kang, Z.; Shao, M. Rhodium Nanoparticles/F-Doped Graphene Composites as Multifunctional Electrocatalyst Superior to Pt/C for Hydrogen Evolution and Formic Acid Oxidation Reaction. *ACS Appl. Mater. Interfaces* **2018**, *10* (39), 33153–33161. https://doi.org/10.1021/acsami.8b09297.

(57) Li, Z.; Chen, Y.; Ji, S.; Tang, Y.; Chen, W.; Li, A.; Zhao, J.; Xiong, Y.; Wu, Y.; Gong, Y.; Yao, T.; Liu, W.; Zheng, L.; Dong, J.; Wang, Y.; Zhuang, Z.; Xing, W.; He, C. T.; Peng, C.; Cheong, W. C.; Li, Q.; Zhang, M.; Chen, Z.; Fu, N.; Gao, X.; Zhu, W.; Wan, J.; Zhang, J.; Gu, L.; Wei, S.; Hu, P.; Luo, J.; Li, J.; Chen, C.; Peng, Q.; Duan, X.; Huang, Y.; Chen, X. M.; Wang, D.; Li, Y. Iridium Single-Atom Catalyst on Nitrogen-Doped Carbon for Formic Acid Oxidation Synthesized Using a General Host–Guest Strategy.

Nat. Chem. **2020**, *12* (8), 764–772. https://doi.org/10.1038/s41557-020-0473-9.

(58) Han, A.; Zhang, Z.; Yang, J.; Wang, D.; Li, Y. Carbon-Supported Single-Atom Catalysts for Formic Acid Oxidation and Oxygen Reduction Reactions. *Small* **2021**, *2004500*, 1–15. https://doi.org/10.1002/smll.202004500.

(59) Adžić, R. R.; Simić, D. N.; Despić, A. R.; Dražić, D. M. Electrocatalysis by Foreign Metal Monolayers: Oxidation of Formic Acid on Platinum. *J. Electroanal. Chem.* **1975**, *65* (2), 587–601. https://doi.org/10.1016/0368-1874(75)85146-X.

(60) Campbell, S. A.; Parsons, R. Effect of Bi and Sn Adatoms on Formic Acid and Methanol Oxidation at Well Defined Platinum Surfaces. *J. Chem. Soc. Faraday Trans.* **1992**, *88* (6), 833–841. https://doi.org/10.1039/FT9928800833.

(61) Kizhakevariam, N.; Weaver, M. J. Structure and Reactivity of Bimetallic Electrochemical Interfaces: Infrared Spectroscopic Studies of Carbon Monoxide Adsorption and Formic Acid Electrooxidation on Antimony-Modified Pt(100) and Pt(111). *Surf. Sci.* **1994**, *310* (1–3), 183–197. https://doi.org/10.1016/0039-6028(94)91383-8.

(62) Yang, Y. Y.; Sun, S. G.; Gu, Y. J.; Zhou, Z. Y.; Zhen, C. H. Surface Modification and Electrocatalytic Properties of Pt(100), Pt(110), Pt(320) and Pt(331) Electrodes with Sb towards HCOOH Oxidation. *Electrochim. Acta* **2001**, *46* (28), 4339–4348. https://doi.org/10.1016/S0013-4686(01)00722-8.

(63) Climent, V.; Herrero, E.; Feliu, J. M. Electrocatalysis of Formic Acid and CO Oxidation on Antimony-Modified Pt(111) Electrodes. *Electrochim. Acta* **1998**, *44* (8–9), 1403–1414. https://doi.org/10.1016/S0013-4686(98)00263-1.

(64) Motoo, S.; Watanabe, M. Electrocatalysis by Ad-Atoms. *J. Electroanal. Chem. Interfacial Electrochem.* **1979**, *98* (2), 203–211. https://doi.org/10.1016/S0022-0728(79)80260-0.

(65) Maciá, M. D.; Herrero, E.; Feliu, J. M. Formic Acid Oxidation on Bi-Pt(1 1 1) Electrode in Perchloric Acid Media. A Kinetic Study. *J. Electroanal. Chem.* **2003**, *554–555* (1), 25–34. https://doi.org/10.1016/S0022-0728(03)00023-8.

(66) Boronat-González, A.; Herrero, E.; Feliu, J. M. Heterogeneous Electrocatalysis of Formic Acid Oxidation on Platinum Single Crystal Electrodes. *Curr. Opin. Electrochem.* **2017**, *4* (1), 26–31. https://doi.org/10.1016/j.coelec.2017.06.003.

(67) Kim, B. J.; Kwon, K.; Rhee, C. K.; Han, J.; Lim, T. H. Modification of Pt Nanoelectrodes Dispersed on Carbon Support Using Irreversible Adsorption of Bi to Enhance Formic Acid Oxidation. *Electrochim. Acta* **2008**, *53* (26), 7744–7750. https://doi.org/10.1016/j.electacta.2008.05.061.

(68) Herrero, E.; Llorca, M. J.; Feliu, J. M.; Aldaz, A. Oxidation of Formic Acid on Pt(100) Electrodes Modified by Irreversibly Adsorbed Tellurium. *J. Electroanal. Chem.* **1995**, *383* (1–2), 145–154. https://doi.org/10.1016/0022-0728(94)03721-E.

(69) Perales-Rondón, J. V.; Busó-Rogero, C.; Solla-Gullón, J.; Herrero, E.; Feliu, J. M. Formic Acid Electrooxidation on Thallium Modified Platinum Single Crystal Electrodes. *J. Electroanal. Chem.* **2017**, *800*, 82–88. https://doi.org/10.1016/j.jelechem.2016.09.020.

(70) Miao, K.; Luo, Y.; Zou, J.; Yang, J.; Zhang, F.; Huang, L.; Huang, J.; Kang, X.; Chen, S. PdRu Alloy Nanoparticles of Solid Solution in Atomic Scale: Outperformance towards Formic Acid Electro-Oxidation in Acidic Medium. *Electrochim. Acta* **2017**, *251*, 588–

594. https://doi.org/10.1016/j.electacta.2017.08.167.

(71) Gasteiger, H. A.; Markovic, N.; Ross, P. N.; Cairns, E. J. METHANOL ELECTROOXIDATION ON WELL-CHARACTERIZED PT-RN ALLOYS. *J. Phys. Chem.* **1993**, *97* (46), 12020–12029. https://doi.org/10.1021/j100148a030.

(72) Ye, W.; Chen, S.; Ye, M.; Ren, C.; Ma, J.; Long, R.; Wang, C.; Yang, J.; Song, L.; Xiong, Y. Pt4PdCu0.4 Alloy Nanoframes as Highly Efficient and Robust Bifunctional Electrocatalysts for Oxygen Reduction Reaction and Formic Acid Oxidation. *Nano Energy* **2017**, *39*, 532–538. https://doi.org/10.1016/j.nanoen.2017.07.025.

(73) Xu, J. B.; Zhao, T. S.; Liang, Z. X. Synthesis of Active Platinum-Silver Alloy Electrocatalyst toward the Formic Acid Oxidation Reaction. *J. Phys. Chem. C* **2008**, *112* (44), 17362–17367. https://doi.org/10.1021/jp8063933.

(74) Sun, D.; Si, L.; Fu, G.; Liu, C.; Sun, D.; Chen, Y.; Tang, Y.; Lu, T. Nanobranched Porous Palladium-Tin Intermetallics: One-Step Synthesis and Their Superior Electrocatalysis towards Formic Acid Oxidation. *J. Power Sources* **2015**, *280*, 141–146. https://doi.org/10.1016/j.jpowsour.2015.01.100.

(75) Kang, Y.; Qi, L.; Li, M.; Diaz, R. E.; Su, D.; Adzic, R. R.; Stach, E.; Li, J.; Murray, C. B. Highly Active Pt 3Pb and Core-Shell Pt 3Pb-Pt Electrocatalysts for Formic Acid Oxidation. *ACS Nano* **2012**, *6* (3), 2818–2825. https://doi.org/10.1021/nn3003373.

(76) Jiang, X.; Fu, G.; Wu, X.; Liu, Y.; Zhang, M.; Sun, D.; Xu, L.; Tang, Y. Ultrathin AgPt Alloy Nanowires as a High-Performance Electrocatalyst for Formic Acid Oxidation. *Nano Res.* **2018**, *11* (1), 499–510. https://doi.org/10.1007/s12274-017-1658-4.

(77) Liang, X.; Liu, B.; Zhang, J.; Lu, S.; Zhuang, Z. Ternary Pd-Ni-P Hybrid Electrocatalysts Derived from Pd-Ni Core-Shell Nanoparticles with Enhanced Formic Acid Oxidation Activity. *Chem. Commun.* **2016**, *52* (74), 11143–11146. https://doi.org/10.1039/c6cc04382h.

(78) Zheng, J.; Zeng, H.; Tan, C.; Zhang, T.; Zhao, B.; Guo, W.; Wang, H.; Sun, Y.; Jiang, L. Coral-like PdCu Alloy Nanoparticles Act as Stable Electrocatalysts for Highly Efficient Formic Acid Oxidation. *ACS Sustain. Chem. Eng.* **2019**, *7* (18), 15354–15360. https://doi.org/10.1021/acssuschemeng.9b02677.

(79) Çögenli, M. S.; Yurtcan, A. B. Catalytic Activity, Stability and Impedance Behavior of PtRu/C, PtPd/C and PtSn/C Bimetallic Catalysts toward Methanol and Formic Acid Oxidation. *Int. J. Hydrogen Energy* **2018**, *43* (23), 10698–10709. https://doi.org/10.1016/j.ijhydene.2018.01.081.

(80) Huang, L.; Liu, M.; Lin, H.; Xu, Y.; Wu, J.; Dravid, V. P.; Wolverton, C.; Mirkin, C. A. Shape Regulation of High-Index Facet Nanoparticles by Dealloying. *Science (80-.).* **2019**, *365* (6458), 1159–1163. https://doi.org/10.1126/science.aax5843.

(81) Yu, Y.; Hu, Y.; Liu, X.; Deng, W.; Wang, X. The Study of Pt@Au Electrocatalyst Based on Cu Underpotential Deposition and Pt Redox Replacement. *Electrochim. Acta* **2009**, *54* (11), 3092–3097. https://doi.org/10.1016/j.electacta.2008.12.004.

(82) Wang, X.; Tang, Y.; Gao, Y.; Lu, T. Carbon-Supported Pd-Ir Catalyst as Anodic Catalyst in Direct Formic Acid Fuel Cell. *J. Power Sources* **2008**, *175* (2), 784–788. https://doi.org/10.1016/j.jpowsour.2007.10.011.

(83) Fu, G. T.; Liu, C.; Zhang, Q.; Chen, Y.; Tang, Y. W. Polyhedral Palladium-Silver Alloy Nanocrystals as Highly Active and Stable Electrocatalysts for the Formic Acid Oxidation Reaction. *Sci. Rep.* **2015**, *5* (August), 1–9. https://doi.org/10.1038/srep13703.

(84) Kibler, L. A.; El-Aziz, A. M.; Hoyer, R.; Kolb, D. M. Tuning Reaction Rates by Lateral Strain in a Palladium Monolayer. *Angew. Chemie - Int. Ed.* **2005**, *44* (14), 2080–2084. https://doi.org/10.1002/anie.200462127.

(85) Hu, S.; Che, F.; Khorasani, B.; Jeon, M.; Yoon, C. W.; McEwen, J. S.; Scudiero, L.; Ha, S. Improving the Electrochemical Oxidation of Formic Acid by Tuning the Electronic Properties of Pd-Based Bimetallic Nanoparticles. *Appl. Catal. B Environ.* **2019**, *254* (February), 685–692. https://doi.org/10.1016/j.apcatb.2019.03.072.

(86) Lin, W. F.; Christensen, P. A.; Hamnett, A. The Electro-Oxidations of Methanol and Formic Acid at the Ru(0001) Electrode as a Function of Temperature: In-Situ FTIR Studies. *Phys. Chem. Chem. Phys.* **2001**, *3* (16), 3312–3319. https://doi.org/10.1039/b102699m.

(87) Orozco, G.; Gutiérrez, C. Adsorption and Electro-Oxidation of Carbon Monoxide, Methanol, Ethanol and Formic Acid on Osmium Electrodeposited on Glassy Carbon. *J. Electroanal. Chem.* **2000**, *484* (1), 64–72. https://doi.org/10.1016/S0022-0728(00)00062-0.

(88) Brimaud, S.; Solla-Gullón, J.; Weber, I.; Feliu, J. M.; Behm, R. J. Formic Acid Electrooxidation on Noble-Metal Electrodes: Role and Mechanistic Implications of PH, Surface Structure, and Anion Adsorption. *ChemElectroChem* **2014**, *1* (6), 1075–1083.

https://doi.org/10.1002/celc.201400011.

(89) Liao, M.; Li, W.; Xi, X.; Luo, C.; Gui, S.; Jiang, C.; Mai, Z.; Chen, B. H. Highly Active Aucore@Ptclustercatalyst for Formic Acid Electrooxidation. *J. Electroanal. Chem.* **2017**, *791*, 124–130. https://doi.org/10.1016/j.jelechem.2017.03.024.

(90) Xu, J. B.; Zhao, T. S.; Liang, Z. X. Carbon Supported Platinum-Gold Alloy Catalyst for Direct Formic Acid Fuel Cells. *J. Power Sources* **2008**, *185* (2), 857–861. https://doi.org/10.1016/j.jpowsour.2008.09.039.

(91) Waszczuk, P.; Barnard, T. M.; Rice, C.; Masel, R. I.; Wieckowski, A. A Nanoparticle Catalyst with Superior Activity for Electrooxidation of Formic Acid. *Electrochem. commun.* **2002**, *4* (7), 599–603. https://doi.org/10.1016/S1388-2481(02)00386-7.

(92) Chen, C. H.; Liou, W. J.; Lin, H. M.; Wu, S. H.; Borodzinski, A.; Stobinski, L.; Kedzierzawski, P. Palladium and Palladium Gold Catalysts Supported on MWCNTs for Electrooxidation of Formic Acid. *Fuel Cells* **2010**, *10* (2), 227–233. https://doi.org/10.1002/fuce.200900117.

(93) Chen, D.; Li, J.; Cui, P.; Liu, H.; Yang, J. Gold-Catalyzed Formation of Core-Shell Gold-Palladium Nanoparticles with Palladium Shells up to Three Atomic Layers. *J. Mater. Chem. A* **2016**, *4* (10), 3813–3821. https://doi.org/10.1039/c5ta10303g.

(94) Siahrostami, S.; Verdaguer-Casadevall, A.; Karamad, M.; Deiana, D.; Malacrida, P.; Wickman, B.; Escudero-Escribano, M.; Paoli, E.; Frydendal, R.; Hansen, T. W.; Chorkendorff, I.; Stephens, I. E. L.; Rossmeisl, J. Enabling Direct H2O2 Production through Rational Electrocatalyst Design. *Nat. Mater.* **2013**, *12* (12), 1137–1143.

https://doi.org/10.1038/nmat3795.

(95) Perales-Rondón, J. V.; Brimaud, S.; Solla-Gullón, J.; Herrero, E.; Jürgen Behm, R.; Feliu, J. M. Further Insights into the Formic Acid Oxidation Mechanism on Platinum: PH and Anion Adsorption Effects. *Electrochim. Acta* **2015**, *180*, 479–485. https://doi.org/10.1016/j.electacta.2015.08.155.

(96) Joo, J.; Uchida, T.; Cuesta, A.; Koper, M. T. M. M.; Osawa, M. Importance of Acid-Base Equilibrium in Electrocatalytic Oxidation of Formic Acid on Platinum. *J. Am. Chem. Soc.* **2013**, *135* (27), 9991–9994. https://doi.org/10.1021/ja403578s.

(97) Swamy, B. E. K.; Vannoy, C.; Maye, J.; Kamali, F.; Huynh, D.; II, B. B. L.; Schell, M. Potential Oscillations in Formic Acid Oxidation in Electrolyte Mixtures: Efficiency and Stability. *J. Electroanal. Chem.* **2009**, *625* (1), 69–74. https://doi.org/10.1016/j.jelechem.2008.10.001.

(98) Yurtcan, A. B. Formic Acid and Methanol Oxidation for Pt/C Catalyst Depending on Rotating Speed, Scan Rate and Concentration. *Brill. Eng.* **2019**, *1* (2), 1–4. https://doi.org/10.36937/ben.2020.002.001.

(99) Chen, X.; Koper, M. T. M. Mass-Transport-Limited Oxidation of Formic Acid on a PdMLPt(100) Electrode in Perchloric Acid. *Electrochem. commun.* **2017**, *82* (August), 155–158. https://doi.org/10.1016/j.elecom.2017.08.002.

(100) Wan, H.; Østergaard, T. M.; Arnarson, L.; Rossmeisl, J. Climbing the 3D Volcano for the Oxygen Reduction Reaction Using Porphyrin Motifs. *ACS Sustain. Chem. Eng.* **2019**, *7* (1), 611–617. https://doi.org/10.1021/acssuschemeng.8b04173.

(101) Sciences, P. The Mechanisms of Oxidation of Formaldehyde and Formic Acid by Ions of Chromium (VI), Vanadium (V) and Cobalt (III). *Proc. R. Soc. London. Ser. A. Math. Phys. Sci.* **1963**, *274* (1359), 480–499. https://doi.org/10.1098/rspa.1963.0145.

(102) Escudero-Escribano, M.; Malacrida, P.; Hansen, M. H.; Vej-Hansen, U. G.; Velazquez-Palenzuela, A.; Tripkovic, V.; Schiotz, J.; Rossmeisl, J.; Stephens, I. E. L.; Chorkendorff, I. Tuning the Activity of Pt Alloy Electrocatalysts by Means of the Lanthanide Contraction. *Science* **2016**, *352* (6281), 73–76. https://doi.org/10.1126/science.aad8892.

(103) Inaba, M.; Quinson, J.; Bucher, J. R.; Arenz, M. On the Preparation and Testing of Fuel Cell Catalysts Using the Thin Film Rotating Disk Electrode Method. *J. Vis. Exp.* **2018**, *2018* (133). https://doi.org/10.3791/57105.

(104) Garsany, Y.; Baturina, O. A.; Swider-Lyons, K. E.; Kocha, S. S.; Garsany, Y.; Baturina, O. A.; Swider-Lyons, K. E.; Kocha, S. S.; Garsany, Y.; Baturina, O. A.; Swider-Lyons, K. E.; Kocha, S. S. Experimental Methods for Quantifying the Activity of Platinum Electrocatalysts for the Oxygen Reduction Reaction. *Anal. Chem.* **2010**, *82* (15), 6321–6328. https://doi.org/10.1021/ac100306c.

(105) Arminio-Ravelo, J. A.; Jensen, A. W.; Jensen, K. D.; Quinson, J.; Escudero-Escribano, M. Electrolyte Effects on the Electrocatalytic Performance of Iridium-Based Nanoparticles for Oxygen Evolution in Rotating Disc Electrodes. *ChemPhysChem* **2019**, *20* (22), 2956–2963. https://doi.org/10.1002/cphc.201900902.

(106) Rodríguez, P.; Solla-Gullón, J.; Vidal-Iglesias, F. J.; Herrero, E.; Aldaz, A.; Feliu, J. M. Determination of (111) Ordered Domains on Platinum Electrodes by Irreversible

Adsorption of Bismuth. *Anal. Chem.* **2005**, *77* (16), 5317–5323. https://doi.org/10.1021/ac050347q.

(107) Choi, M.; Ahn, C. Y.; Lee, H.; Kim, J. K.; Oh, S. H.; Hwang, W.; Yang, S.; Kim, J.; Kim, O. H.; Choi, I.; Sung, Y. E.; Cho, Y. H.; Rhee, C. K.; Shin, W. Bi-Modified Pt Supported on Carbon Black as Electro-Oxidation Catalyst for 300 W Formic Acid Fuel Cell Stack. *Appl. Catal. B Environ.* **2019**, *253* (April), 187–195. https://doi.org/10.1016/j.apcatb.2019.04.059.

(108) Jain, A.; Ong, S. P.; Hautier, G.; Chen, W.; Richards, W. D.; Dacek, S.; Cholia, S.; Gunter, D.; Skinner, D.; Ceder, G.; Persson, K. A. Commentary: The Materials Project: A Materials Genome Approach to Accelerating Materials Innovation. *APL Mater.* **2013**, *1* (1), 011002. https://doi.org/10.1063/1.4812323.

(109) Beermann, V.; Gocyla, M.; Kühl, S.; Padgett, E.; Schmies, H.; Goerlin, M.; Erini, N.; Shviro, M.; Heggen, M.; Dunin-Borkowski, R. E.; Muller, D. A.; Strasser, P. Tuning the Electrocatalytic Oxygen Reduction Reaction Activity and Stability of Shape-Controlled Pt-Ni Nanoparticles by Thermal Annealing -Elucidating the Surface Atomic Structural and Compositional Changes. *J. Am. Chem. Soc.* **2017**, *139* (46), 16536–16547. https://doi.org/10.1021/jacs.7b06846.

(110) Hjorth Larsen, A.; JØrgen Mortensen, J.; Blomqvist, J.; Castelli, I. E.; Christensen, R.; Dułak, M.; Friis, J.; Groves, M. N.; Hammer, B.; Hargus, C.; Hermes, E. D.; Jennings, P. C.; Bjerre Jensen, P.; Kermode, J.; Kitchin, J. R.; Leonhard Kolsbjerg, E.; Kubal, J.; Kaasbjerg, K.; Lysgaard, S.; Bergmann Maronsson, J.; Maxson, T.; Olsen, T.; Pastewka, L.; Peterson, A.; Rostgaard, C.; SchiØtz, J.; Schütt, O.; Strange, M.; Thygesen, K. S.;

Vegge, T.; Vilhelmsen, L.; Walter, M.; Zeng, Z.; Jacobsen, K. W. The Atomic Simulation Environment - A Python Library for Working with Atoms. *Journal of Physics Condensed Matter*. {IOP} Publishing June 2017, p 273002. https://doi.org/10.1088/1361-648X/aa680e.

(111) Enkovaara, J.; Rostgaard, C.; Mortensen, J. J.; Chen, J.; Dułak, M.; Ferrighi, L.; Gavnholt, J.; Glinsvad, C.; Haikola, V.; Hansen, H. A.; Kristoffersen, H. H.; Kuisma, M.; Larsen, A. H.; Lehtovaara, L.; Ljungberg, M.; Lopez-Acevedo, O.; Moses, P. G.; Ojanen, J.; Olsen, T.; Petzold, V.; Romero, N. A.; Stausholm-Møller, J.; Strange, M.; Tritsaris, G. A.; Vanin, M.; Walter, M.; Hammer, B.; Häkkinen, H.; Madsen, G. K. H.; Nieminen, R. M.; Nørskov, J. K.; Puska, M.; Rantala, T. T.; Schiøtz, J.; Thygesen, K. S.; Jacobsen, K. W. Electronic Structure Calculations with GPAW: A Real-Space Implementation of the Projector Augmented-Wave Method. *J. Phys. Condens. Matter* **2010**, *22* (25). https://doi.org/10.1088/0953-8984/22/25/253202.

(112) Wellendorff, J.; Lundgaard, K. T.; Møgelhøj, A.; Petzold, V.; Landis, D. D.; Nørskov, J. K.; Bligaard, T.; Jacobsen, K. W. Density Functionals for Surface Science: Exchange-Correlation Model Development with Bayesian Error Estimation. *Phys. Rev. B - Condens. Matter Mater. Phys.* **2012**, *85* (23), 32–34. https://doi.org/10.1103/PhysRevB.85.235149.
